# Lipidomimetic Compounds Act as HIV-1 Entry Inhibitors by Altering Viral Membrane Structure

**DOI:** 10.3389/fimmu.2018.01983

**Published:** 2018-09-04

**Authors:** Jon Ander Nieto-Garai, Bärbel Glass, Carmen Bunn, Matthias Giese, Gary Jennings, Beate Brankatschk, Sameer Agarwal, Kathleen Börner, F. Xabier Contreras, Hans-Joachim Knölker, Claudia Zankl, Kai Simons, Cornelia Schroeder, Maier Lorizate, Hans-Georg Kräusslich

**Affiliations:** ^1^Departamento de Bioquímica y Biología Molecular, Instituto Biofisika (CSIC, UPV/EHU), Universidad del País Vasco, Bilbao, Spain; ^2^Department of Infectious Diseases, Virology, Universitätsklinikum Heidelberg, Heidelberg, Germany; ^3^JADO Technologies, Dresden, Germany; ^4^Membrane Biochemistry Group, Paul-Langerhans-Institute Dresden, Helmholtz Zentrum München at the University Hospital and Faculty of Medicine Carl Gustav Carus, Dresden, Germany; ^5^Department of Chemistry, Technische Universität Dresden, Dresden, Germany; ^6^Ikerbasque, Basque Foundation for Science, Bilbao, Spain; ^7^Max Planck Institute of Molecular Cell Biology and Genetics, Dresden, Germany; ^8^Department of Anatomy, Medical Faculty Carl-Gustav-Carus, Technische Universität Dresden, Dresden, Germany

**Keywords:** lipidomimetics, HIV-1 envelope, lipid raft modulation, laurdan, membrane order, HIV fusion inhibitors, phosphatidylserine

## Abstract

The envelope of Human Immunodeficiency Virus type 1 (HIV-1) consists of a liquid-ordered membrane enriched in raft lipids and containing the viral glycoproteins. Previous studies demonstrated that changes in viral membrane lipid composition affecting membrane structure or curvature can impair infectivity. Here, we describe novel antiviral compounds that were identified by screening compound libraries based on raft lipid-like scaffolds. Three distinct molecular structures were chosen for mode-of-action studies, a sterol derivative (J391B), a sphingosine derivative (J582C) and a long aliphatic chain derivative (IBS70). All three target the viral membrane and inhibit virus infectivity at the stage of fusion without perturbing virus stability or affecting virion-associated envelope glycoproteins. Their effect did not depend on the expressed envelope glycoproteins or a specific entry route, being equally strong in HIV pseudotypes carrying VSV-G or MLV-Env glycoproteins. Labeling with laurdan, a reporter of membrane order, revealed different membrane structure alterations upon compound treatment of HIV-1, which correlated with loss of infectivity. J582C and IBS70 decreased membrane order in distinctive ways, whereas J391B increased membrane order. The compounds' effects on membrane order were reproduced in liposomes generated from extracted HIV lipids and thus independent both of virion proteins and of membrane leaflet asymmetry. Remarkably, increase of membrane order by J391B required phosphatidylserine, a lipid enriched in the HIV envelope. Counterintuitively, mixtures of two compounds with opposite effects on membrane order, J582C and J391B, did not neutralize each other but synergistically inhibited HIV infection. Thus, altering membrane order, which can occur by different mechanisms, constitutes a novel antiviral mode of action that may be of general relevance for enveloped viruses and difficult to overcome by resistance development.

## Introduction

Human immunodeficiency virus type 1 (HIV-1) is an enveloped retrovirus, which infects CD4-positive human cells. HIV-1 morphogenesis at the plasma membrane of the infected cell is driven by the viral Gag polyprotein whose N-terminal MA (matrix) domain interacts with phosphatidylinositol (4,5) bisphosphate (PI(4,5)P_2_) [reviewed in (1)]. Since retroviruses do not encode lipid-synthesizing enzymes, their lipid envelope composition depends on the membrane through which the virus buds (2, 3). However, viral membrane composition may differ from the donor cell membrane if virus assembly occurs at membrane subdomains or involves lipid sorting. Early studies of HIV-1 lipid composition indicated significant differences between the viral membrane and the host cell plasma membranes (4, 5). This observation was later confirmed by more detailed analyses of the entire viral lipidome. The HIV-1 membrane was shown to be significantly enriched in phosphatidylserine (PS), sphingomyelin, hexosylceramide and saturated phosphatidylcholine species when compared to the host cell plasma membrane (6–8). Overall, the HIV-1 lipid composition is typical of lipid rafts (6). Moreover, labeling HIV-1 with the order-sensing dye laurdan revealed a liquid-ordered (l_o_) structure of the viral envelope (9).

The intrinsic properties of the viral membrane, as well as its lipid composition, have been shown to be of importance for infectivity. Cholesterol-depleting agents (β-cyclodextrin and statins) (10–13) or cholesterol-binding compounds (amphotericin B methyl ester) (14) as well as inhibition of sphingomyelin biosynthesis (6, 15, 16) strongly reduced HIV-1 infectivity, indicating an important contribution of its raft-like membrane lipid composition and/or structure to viral infection. A similar effect was observed when ceramide levels were increased in the viral membrane (17) or upon addition of a compound (GT11), which leads to higher dihydrosphingomyelin levels (18), an unusual lipid enriched in the viral membrane (6). Based on the concept of inverted cone-shaped lipids as fusion inhibitors (19), synthetic rigid amphipathic fusion inhibitors (RAFIs) have been designed as potential antivirals (20). These compounds insert into the viral membrane and promote positive curvature, thus increasing the energy barrier for fusion. RAFIs were shown to inhibit fusion of several unrelated enveloped viruses.

Here, we performed a screen of lipidomimetic compounds, the majority of which resembling raft lipids, for their capacity to alter the membrane of HIV-1 and interfere with viral infectivity. Several compounds structurally related to cholesterol, sphingosine or aliphatic lipids with long-chain fatty acids inhibited HIV-1 infection at the stage of entry. Similar inhibition was observed when HIV-1 was pseudotyped with heterologous envelope proteins, indicating that the effect was independent of the initial entry pathway and the envelope proteins mediating it. Incorporation of the compounds into the viral membrane inhibited viral membrane fusion, induced changes in viral membrane order and subtle shifts in particle buoyant density. Thus, altering virion membrane structure by lipid-active compounds may be a promising approach for inhibiting HIV.

## Materials and methods

### Cell culture and virus purification

293T and TZM reporter cells (21) and DFJ8 cells (22) were kept in Dulbecco's modified Eagle's medium (DMEM), MT-4 cells (23) were kept in RPMI 1,640 medium. Both media were supplemented with 10% heat inactivated fetal calf serum (FCS), penicillin, streptomycin, 4 mM glutamine, and 10 mM Hepes. Cell cultures were maintained at 37°C and 5% CO_2_. All investigated HIV strains and constructs are listed in Table S1 in the Supplementary Material. For virus production, MT-4 cells were infected with HIV-1 strain NL4-3 (24), and virus was harvested from cocultures of infected and uninfected cells before cytopathic effects were observed (25). 293T cells were transfected with the proviral plasmid pNL4-3 (24) or with pCHIV (26) by calcium phosphate precipitation. For generation of pseudotyped particles, cells were co-transfected with pNL4-3 carrying a deletion of the envelope gene and a plasmid expressing either the G glycoprotein of Vesicular Stomatitis Virus (VSV) (27) or the envelope proteins of Friend ecotropic Murine Leukemia Virus (MLV) (28) at a molar ratio 1:2. HIV-1 purification was performed essentially as described (25, 29). Briefly, medium was harvested, cleared by filtration, and particles were concentrated by ultracentrifugation through a cushion of 20% (w/w) sucrose. Concentrated HIV-1 was further purified by velocity gradient centrifugation on an Optiprep gradient (Axis-Shield; Oslo, Norway). This step largely removes exosomes and membrane vesicles. The visible virus fraction was collected and concentrated by centrifugation. The final pellet was resuspended in 150 mM NaCl, 10 mM Hepes pH 7.4, rapidly frozen in liquid nitrogen and stored at −80°C. The particle concentration was determined by enzyme-linked immunosorbent assay (ELISA) of p24. Inactivation of infectious HIV-1 was performed by incubating the virus with 5 mM AT-2 (2,2'-dithiodipyridine; aldrithiol-2; Sigma; St. Louis, MO, USA) for 1 h at 37°C with gentle stirring as described (30). Successful inactivation was controlled by culturing inactivated samples for 10 days with highly susceptible C8166 cells. In the case of adeno-associated virus (AAV) purification, standard triple transfection, and caesium chloride (CsCl) density gradient purification procedures were used (31).

### Chemistry

The preparation of 3ß-amino-28-methoxylupene is described in (32). J391B (3α-amino-28-methoxylupene) was synthesized by GVK Bio (Hyderabad, India) following the identical procedure for the ß-anomer. The purity was > 98% by high-performance liquid chromatography (HPLC). The synthesis of J582C (Oxazolin 200) was described (33) with a purity of 99.2% by HPLC. IBS70 (STOCK1S-60139) and IBS95 (STOCK3S-53354) were purchased from Interbioscreen Ltd. (http://www.ibscreen.com).

### Screening

Each compound was screened in duplicate, and each screen was repeated. Compound stock solutions at 2 mM were in glass vials. 100 μl purified HIV-1_NL4−3_ (250 μl/well; 0.5–0.75 μg/ml) was incubated for 30 min at 400 rpm and 37°C on the thermomixer (Eppendorf) with the compounds at 20 μM and 1% FCS in a 96-well glass-coated V-bottom plate (LabHut) and then diluted 1:10 into MT-4 cell suspension culture. 180 μl MT-4 cells were seeded into 96-well plates (CORNING, Poly-D-Lysine surface) at a cell density of 10^5^/well. 20 μl (25–35 ng p24) virus-compound mixture was added to the cells, mixed by pipetting and incubated at 37°C. 18 h p.i. DNA was extracted and subjected to real-time PCR. DNA was isolated using QIAamp 96 DNA Blood Kit and vacuum extraction according to supplier's instruction. Real time PCR was performed with QuantiTect SYBR Green PCR Master MIX (Qiagen): 20 μl reaction mix; twin.tec real-time PCR plates 96 (skirted) and optical caps for RT-PCR; 10 μl master mix plus primers (TIP Molbiol, Berlin) at a concentration of 10 μM each +8.8 μl DNA template. Program: 1x 15 min 95°C; 40x 15 s 95°C; 30 s 60°C; 30 s 72°C.

### Compound treatment of virus particles and cells

MT-4 cells were seeded in poly-D-lysine 96-well plates (CORNING, Poly-D-Lysine surface) and TZM cells were seeded in glass 96-well plates (Costar). Stocks of purified HIV-1_NL43_ were incubated with the different compound concentrations or DMSO as solvent control, in glass-coated plates (Costar) for 30 min at 37°C in RPMI or DMEM medium containing 0.1% FCS. Subsequently, 50 μl virus-compound suspension was diluted into 150 μl medium containing 0.1% FCS (final p24 amounts 25–35 ng) and used to infect target cells for 2 h. Following 2 h exposure, cells were washed and cultivated for 2 more days in complete DMEM or RPMI media. For pretreatment of cells, compounds at the indicated concentrations or DMSO as solvent control were incubated in glass-coated 96-well plates (250 μl/well) for 30 min at 37°C in DMEM containing 0.1% FCS. Subsequently, 100 μl of each compound suspension was added to the target cells for 30 min at 37°C followed by addition of untreated HIV-1 (25–35 ng p24) in 50 μl of medium with 0.1% FCS. Following 2 h exposure, cells were washed and cultivated for 2 more days in complete DMEM followed by infectivity readout as above. A similar procedure was done for simultaneous virus and compound addition. Cells were pre-washed with media containing 0.1% FCS, and compounds at the indicated concentrations or DMSO as solvent control were incubated in glass-coated 96-well plates (250 μl/well) for 30 min at 37°C in DMEM containing 0.1% FCS. Afterwards 100 μl of compounds was added to the cells and immediately followed by adding untreated HIV-1 (25–35 ng p24) in 50 μl of medium with 0.1% FCS for 2 h. Following a washing step, cells were cultivated for 2 more days in complete DMEM and scored for viral infectivity.

### Infectivity and luciferase reporter assay

To determine the effect of the compounds in virus infection, intracellular capsid (CA) staining was performed. MT-4 cells were seeded in poly-D-lysine 96-well plates (CORNING, Poly-D-Lysine surface). Cells were infected, as explained in the previous section, with different amounts of compound-pretreated virus for 2 h, followed by cultivation in medium containing 10% FCS for 2 more days. Subsequently, cells were fixed with 4% paraformaldehyde and permeabilized for immunostaining. HIV-1 infected cells were identified by automated microscopic readout following staining with a phycoerythrin-conjugated antibody against the viral p24 CA protein (KC57-RD1; Beckman Coulter, Inc. Fullerton, USA). For each well the microscope takes 16 measurements. In case of AAV infection, TZM cells were seeded in glass 96-well plates (Costar). Afterwards virus-compound mixtures in medium containing 0.1% FCS were added to TZM cells for 2 h, followed by cultivation in medium containing 10% FCS for 2 more days. To quantify AAV-infected cells, the encoded mCherry reporter was detected by automated microscopy 48 h after infection. Images were acquired via fluorescence microscopy and then automatically analyzed using proprietary software. The infectivity of compound-treated HIV-1 was determined on TZM-bl reporter cells as described (34) with some modifications. TZM-bl reporter cells contain a luciferase gene under a promoter activated by the viral Tat protein. Upon infection and viral gene expression, the production of viral Tat protein induces luciferase gene expression, which enables the quantification of infection by measuring luciferase activity (35). TZM-bl cells (1.2 × 10^4^ cells/well) were seeded one day before infection in a 96-well plate and were infected with compound-treated virus at desired concentrations as explained above. At 48 h post-infection, cells were lysed and luciferase activity was measured in the lysates as described by the manufacturer using the Promega Steady Glo kit and a microplate luminometer (Luminoskan Ascent; Thermo Labsystems, MA, USA). Uninfected cells were cultivated in the presence of compounds at the identical concentrations used in the infection assays, or in the presence of solvent alone (reference control). Following 2 h compound exposure, cells were washed and cultivated for 2 more days, at which time the cytotoxicity was determined by quantifying the amount of a formazan product metabolized by viable cells from the 3-(4,5-dimethylthiazol-2-yl)-2,5-diphenyltetrazolium bromide (MTT) solution (Sigma) as reported (36). Alternatively, compounds were present for 2 days before the MTT assay.

### Entry assays

Standard HIV fusion assays were performed as described (37). HIV-1 particles carrying a Vpr-β-lactamase (Vpr-BlaM) fusion protein were obtained by co-transfection of 293T cells with pNL4-3 or plasmids for HIV pseudotype production and plasmid pMM310 (38) encoding the Vpr-BlaM fusion protein (5 μg pMM310: 15 μg pNL4-3). Particles were harvested, concentrated, and treated with the respective compounds as described above. The virus-compound mixture was added to cells and cells were subsequently washed once with CO_2_-independent medium (Invitrogen), 70 μl of CCF2 β-lactamase loading solution (Invitrogen; prepared according to the manufacturer's instructions) was added and incubation was continued for 17 h at room temperature. Relative fluorescence intensities [excitation wavelength 409 nm, emission wavelengths 447 nm (blue) and 512 nm (green)] were recorded using a TECAN Safire instrument. After subtraction of background from unstained cells at the respective emission wavelength, the ratio of emission intensities at 447/512 nm was calculated.

### Sucrose-density equilibrium gradient centrifugation and western blot analysis

HIV-1_NL4−3_ or non-infectious HIV-like particles derived by transfection of pCHIV (1–3 μg p24) were incubated with the respective compound, solvent (DMSO; 0.35%) or Triton X-100 (TX-100) (0.5%). Subsequently, the particle suspensions were loaded onto a 20–60% linear sucrose gradient. After ultracentrifugation at 44.000 rpm for 16 h at 4°C in a SW60 rotor, 20 fractions of 200 μl each were carefully collected from top to bottom and the p24 concentration was analyzed as described (39). Their sucrose density was calculated from refractive indices determined with a refractometer (Abbe, Carl Zeiss). Carefully prepared sucrose solutions were used to build the gradient and as standards in the refractometer. For this purpose a sucrose gradient was run placing loading buffer instead of particle suspension on top of the gradient. After ultracentrifugation 20 fractions of 200 μl each were carefully collected from top to bottom and their refractive index was measured. Sucrose density in g/cm^3^ was calculated from the refractive index of the standards. For stability analysis, purified HIV-1 or HIV-like particles (3 μg p24) were exposed to compounds or solvent (DMSO; 0.35%) for 30 min at 37°C, pelleted through a 20% sucrose cushion by ultracentrifugation (32.000 rpm, 4°C, 2 h) and re-suspended in 25 μl SDS-PAGE sample buffer for subsequent analysis by Western blotting. Briefly, samples were boiled in sodium dodecyl sulfate (SDS) sample buffer, separated by 12.5% SDS-polyacrylamide gel electrophoresis (SDS-PAGE), and transferred onto a polyvinylidene difluoride (PVDF) membrane. After incubation with mouse monoclonal antibody against the gp41 trans-membrane glycoprotein 1:2000 [Chessie 8; (40)]; rabbit anti-MA, 1:5000; and sheep anti-p24 (CA), 1:5000, detection was carried out with a LiCoR Odyssey system using as secondary antibodies donkey anti-rabbit 700; donkey anti-sheep 800 and donkey anti-mouse 800, 1:20.000 (LiCoR).

### HIV-1 laurdan staining and analysis of labeled particles

Optiprep-purified HIV-1 particles were incubated for 10 min at room temperature with 5 μM laurdan (Molecular Probes, Eugene, OR). Labeled HIV-1 particles were subsequently purified by ultracentrifugation as described (9). In the case of compound treatment, laurdan labeled HIV-1 particles were incubated with different amounts of lipidomimetics at 37°C for 30 min under gentle stirring. Subsequently, viral particles were collected by ultracentrifugation for 2 h through a 20% sucrose cushion in a SW60 rotor at 32.000 rpm. Particles were carefully resuspended in 150 mM NaCl, 10 mM Hepes pH 7.4 and analyzed by fluorescence spectroscopy. All fluorescence measurements were made using an SLM Aminco series 2 (Spectronic Instruments, Rochester, NY) spectrofluorimeter as described (9). To quantify changes in the laurdan emission spectrum, generalized polarization (GP) values were calculated: GP = (I_B_-I_R_)/(I_B_+I_R_), where I_B_ (at 440 nm) and I_R_ (at 490 nm) correspond to the intensities at the blue and red edges of the emission maxima, respectively (41, 42).

### Lipid extraction, production of lipid vesicles and intervesicular MPER lipid mixing assay

Lipid extraction was performed as described (6). Large unilamellar vesicles (LUV) were prepared following the extrusion method (43). Laurdan-labeled LUV at a concentration of 30 μM, as described (9), were treated with specified amounts of lipidomimetics in PBS containing 0.1% FCS for 30 min at 37°C under continuous stirring, followed by determination of GP profiles as above. The following lipids were purchased from Avanti lipids: 1-palmitoyl-2-oleoyl-*sn*-glycero-3-phosphocholine (POPC); 1,2-dipalmitoyl-*sn*-glycero-3-phosphocholine (DPPC); 1,2-dioleoyl-*sn*-glycero-3-phosphate (DOPA); 1,2-dioleoyl-*sn*-glycero-3-phospho-L-serine (DOPS); 1,2-dioleoyl-*sn*-glycero-3-phosphoglycerol (DOPG); cholesterol (CHO); brain sphingomyelin (SM); 1-palmitoyl-2-hydroxy-sn-glycero-3-phosphocholine (LPC); L-α-phosphatidylethanolamine-N-(7-nitro-2-1,3-benzoxadiazol-4-yl) (N-NBD-PE); L-α-phosphatidylethanolamine-N-(lissamine rhodamine B sulfonyl) (N-Rho-PE).

Membrane lipid mixing was monitored in an SLM Aminco series 2 (Spectronic Instruments, Rochester, NY) spectrofluorimeter using a resonance energy transfer assay. The assay is based on the dilution of N-NBD-PE and N-Rho-PE as described (44) with some modifications. Briefly, N-Rho-PE acts as a quencher of the N-NBD-PE fluorescence when both are present in a vesicle membrane at sufficient concentration. Upon dilution (e.g., by fusion of labeled with unlabeled vesicles), the fluorescence of N-NBD-PE is dequenched and becomes detectable. Labeled 10 μM POPC:POPS:SM:Chol (18%:15%:33%:33%) LUVs (NBD/Rho LUV) were incubated with different compound concentrations or LPC as fusion inhibition control for 15 min at 25°C with continuous stirring. Afterwards, 40 μM of unlabeled LUV were added for 20 more min. Once the baseline signal stabilized (0% fusion), the system was considered equilibrated. Then, 0.5 μM of membrane-proximal external region (MPER) peptide was added to launch the fusion reaction and incubation continued for 15 min at 25°C. Intervesicular lipid mixing was measured as an increase of fluorescence of N-NBD-PE caused by fusion of the labeled and unlabeled vesicle membranes. Finally, in order to obtain the 100% fusion value, 0.1% of Triton X-100 was added. The fluorescence baseline before addition of MPER was defined as 0% fusion and maximal fluorescence following final detergent addition was defined as 100% of fusion.

### Statistics

Experimental groups were compared and significance determined by analysis of variance and Tukey test using SigmaPlot. Data are represented as means with standard deviation (± SD).

## Results

### Compound screening

**Figure 1:**
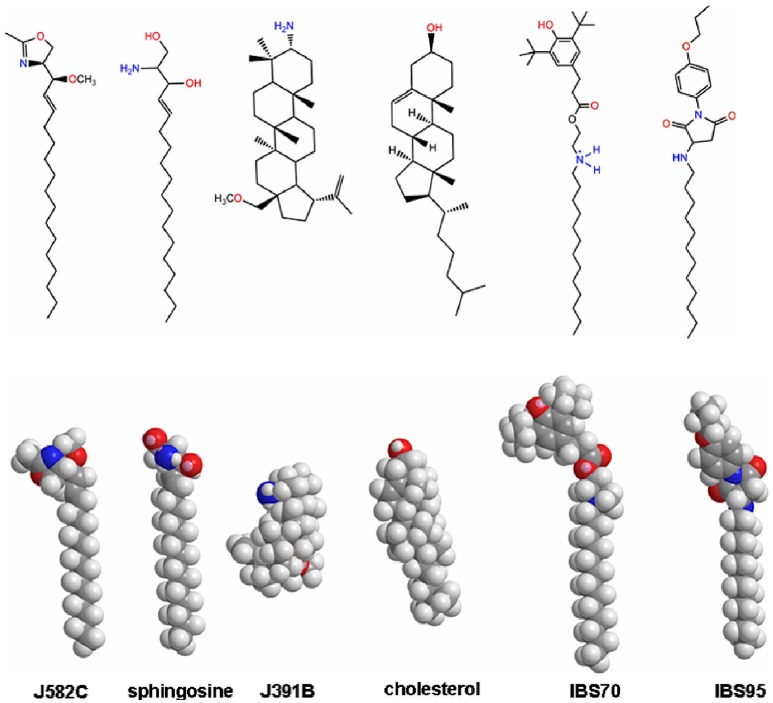
Molecular modeling of the 3D structures of lipidomimetic compounds in comparison to natural lipids. Lowest energy structures in vacuum were computed for relevant compounds and the analogous natural lipids using CS Chem3D Ultra software employing the MM2-force field and the steepest-descent algorithm. Minimum RMS gradient was set to 0.1; minimum and maximum moved to 0.00001 and 1.0, respectively.

Based on prior screening of hydrophobic compounds for a variety of indications where membrane raft disruption was expected to be disease-modulating (45–47) and taking into account published anti-HIV approaches targeting the virus envelope (20, 48), we screened 695 compounds from the proprietary JADO Technologies raft modulator library, combined with selected compounds from commercial libraries of amphiphiles and lipidomimetics. The emphasis was on raft lipid-like scaffolds with 30% of all compounds being sterol-derivatives, 10% ceramide and sphingosine-like molecules, and 20% long (≥ 14 C-atoms) aliphatic main chain- or fatty acid-like (alkyl) phospholipids. Purified HIV-1 (cp. HIV strains and constructs listed in Table S1 in the Supplementary Material) was incubated with compounds at 20 μM in the presence of 1% serum for 30 min at 37°C and subsequently added to target cells. The infection process was terminated after 18 h and DNA was extracted for real-time PCR analysis of viral reverse transcription products. 214 compounds inhibited infection > 90% (IC_90_) at this concentration. After further testing of these compounds at 2 μM, 16 hits achieved > 90% inhibition. Dose-effect relations were recorded for these compounds. Three lipidomimetics were selected from the original 16 hits for mode of action studies, based on the following qualitative criteria: (1) structural diversity, i.e., distinct structural scaffolds similar to different classes of raft lipids; (2) strongest anti-HIV effect during screening and in confirmatory assays; (3) low toxicity in various preliminary assays and cell lines. We chose three structurally distinct compounds (Figure 1), a sterol derivative (J391B), a sphingosine derivative (J582C) and a long chain aliphatic lipidomimetic with an unnatural head group (IBS70). A structure that proved inactive in the screen (IBS95), while exhibiting generic characteristics of aliphatic inhibitory compounds, was selected as a negative control.

### Lipidomimetics inhibit HIV-1 infection

**Figure 2:**
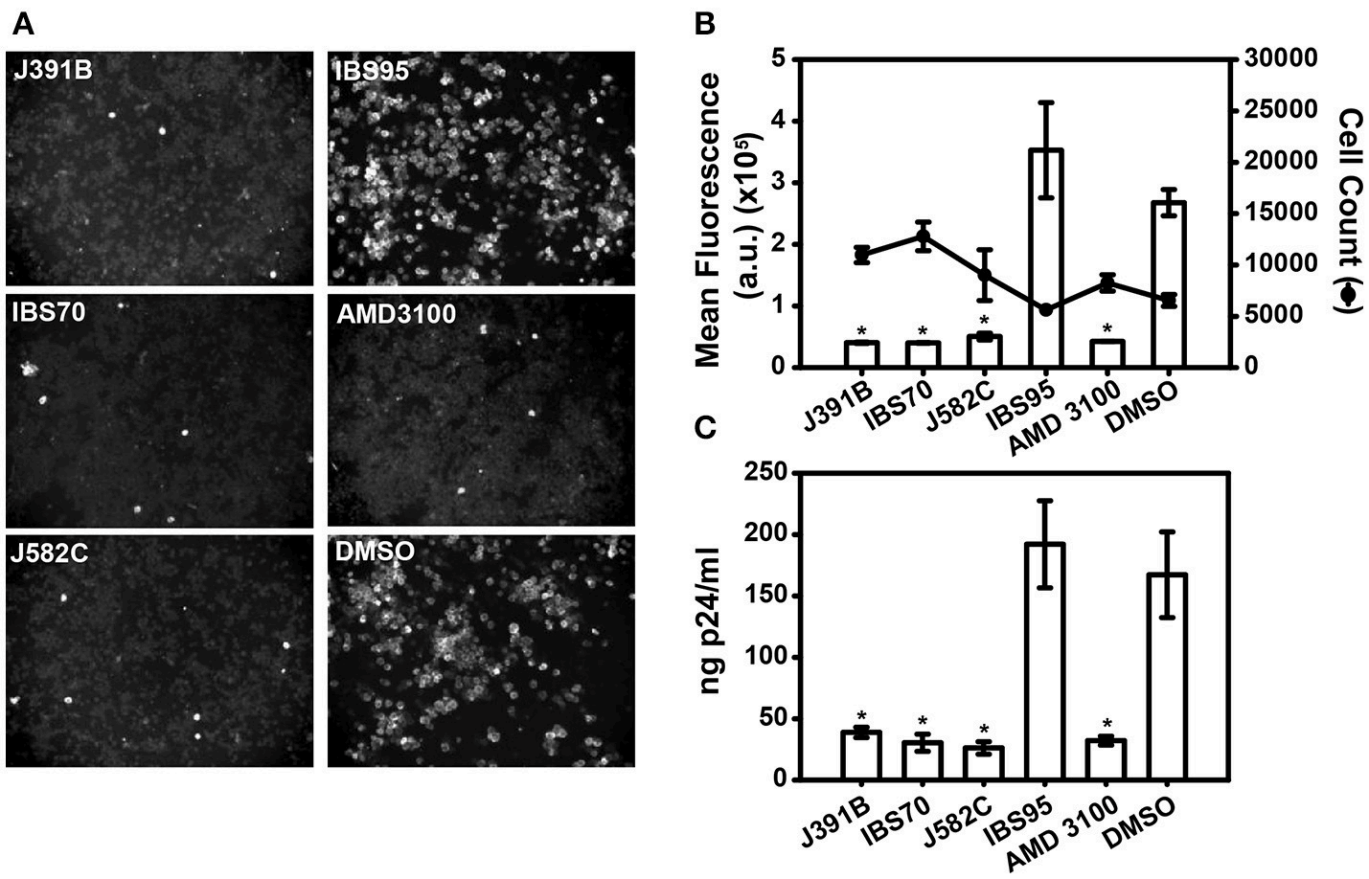
Lipidomimetics affect HIV-1 infectivity. Immunofluorescence microscopy of intracellular p24 in infected MT-4 cells. HIV-1 was pretreated with 6 μM J391B, 2 μM IBS70, 20 μM J582C, 7 μM IBS95, 1% DMSO, or 0.5 μM AMD3100 (as indicated in each panel) for 30 min and subsequently added to MT-4 cells for 2 h. Cells were stained 30 h p.i. **(B)** Infectivity with or without compound treatment was automatically quantified from immunofluorescence images as shown in **(A)** by determining the mean fluorescence of infected MT-4 cells. The effect of compound or solvent treatment on cell number was quantified in parallel (right axis). Data represent the mean ± SD of two replicate experiments with 16 replicas each; ^*^represents a significant (*p* < 0.01) decrease when compared to the DMSO control. **(C)** Infectivity of compound-treated HIV-1 determined by quantitation of progeny virus. HIV-1 was pretreated as described in **(A)** and used to infect MT-4 cells. Released virus at 30 h p.i. was quantitated by p24 ELISA. Data represent the mean ± SD of two replicate experiments with four replicas each; ^*^represents a significant (*p* < 0.01) decrease when compared to the DMSO control.

**Figure 3:**
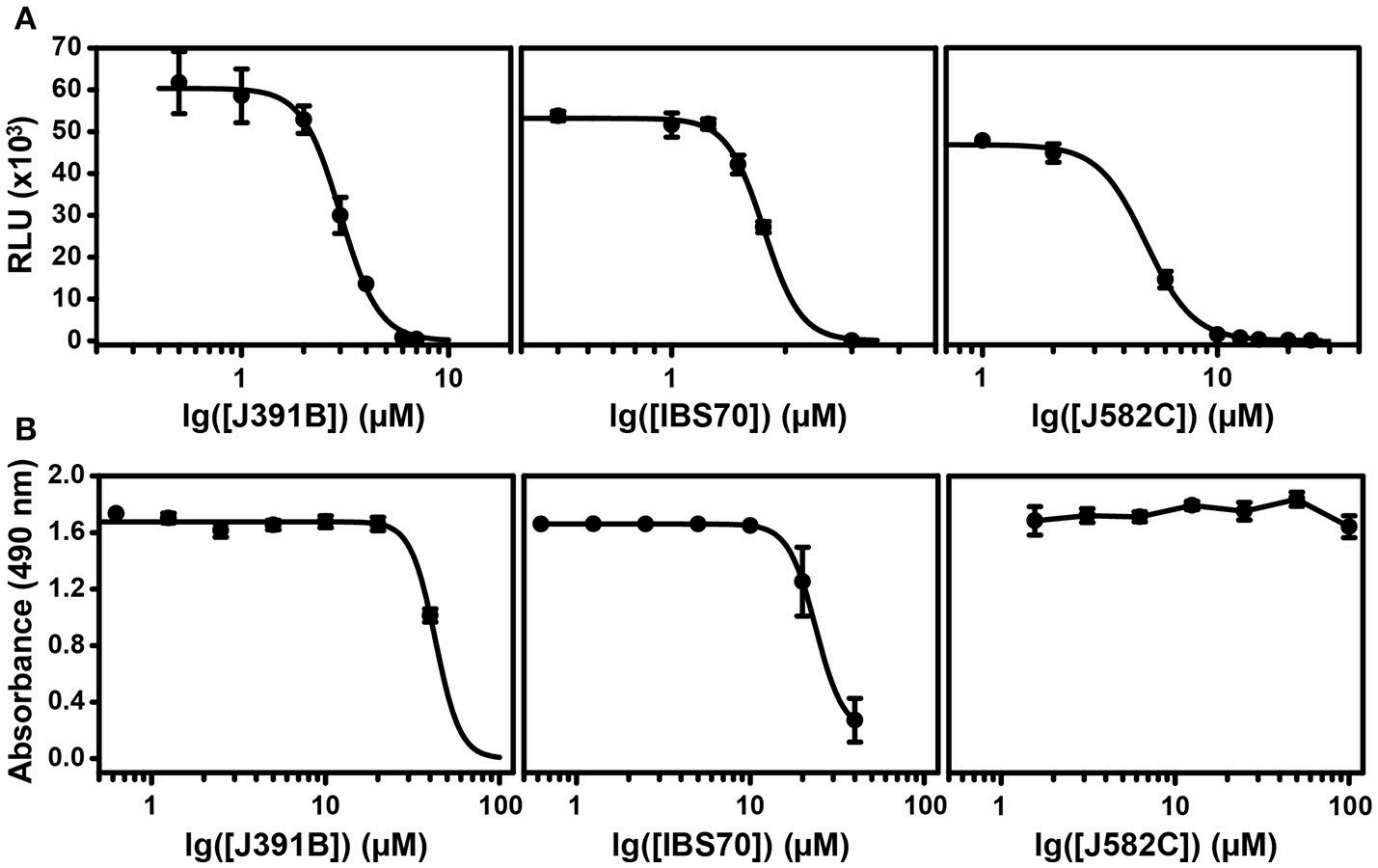
Determination of IC_50_ and CC_50_ of each compound. **(A)** Titration of inhibitory effects. HIV-1 was pretreated with compounds at different concentrations followed by infection of TZM-bl cells. TZM-bl reporter cells were harvested 42–45 h p.i. and luciferase activity induced by newly produced HIV-1 Tat was measured and is shown as relative light units (RLU). RLU values are plotted against compound concentration in a semi-logarithmic way. Data represent the mean ± SD of four replicate experiments with nine replicas each. **(B)** Determination of the 50% cytotoxic concentration of lipidomimetics. TZM-bl cells were incubated for 48 h in the presence of the compounds as indicated in each panel. The CC_50_ for each compound was calculated from the dose-effect relation; mean values were 40 μM for J391B, 25 μM for IBS70 and >100 μM for J582C. Data represent the mean ± SD of four replicate experiments with four replicas each.

To investigate the effects of these lipidomimetics on HIV-1 infectivity, MT-4 T-cells were infected with the prototypic CXCR4-tropic strain HIV-1_NL4−3_, which was pretreated or not with the compounds at previously determined concentrations. Infectivity was monitored by microscopic readout after staining for the viral p24 (CA) antigen (Figure 2A). Immunofluorescence images randomly taken from infected MT-4 cells showed that the three active compounds at their IC_90_s, but not the inactive control, inhibited HIV-1 infection to a similar extent as the CXCR4 co-receptor antagonist AMD3100 [0.5 μM; (49)] that served as positive control. Automated microscopic readout allowed quantitation of the inhibitory effects (Figure 2B), confirming inhibition of HIV-1 infection at these concentrations. Moreover, virus production was strongly reduced when cells were infected with HIV-1 that had been pretreated with the respective compounds (Figure 2C), demonstrating a sustained effect. Compound toxicity was examined by the standard MTT viability assay (36) (Figure S1) and by quantifying cell counts upon automated microscopy (Figure 2B); no toxic effects were observed at the relevant concentrations. Concentration-dependent inhibition of HIV-1 replication and potential cytotoxic effects were further investigated by titration experiments (Figure 3). The 50% inhibitory concentration (IC_50_) was 2.5 μM for J391B, 1.7 μM for IBS70, and 4.6 μM for J582C in TZM cells (Figure 3A). The maximum tolerated concentrations in TZM cells were 20 μM for J391B, 10 μM for IBS70, and (at least) 100 μM for J582C, and their 50% cytotoxic concentrations (CC_50_) were 40 μM for J391B, 25 μM for IBS70, and > 100 μM for J582C (Figure 3B). Because the available stock solution of J582C was 10 mM concentrations above 100 μM could not be tested. The resulting selectivity indices were 15.8, 14.2, and > 21 for J391B, IBS70, and J582C, respectively.

### Lipidomimetics target the virion membrane

**Figure 4:**
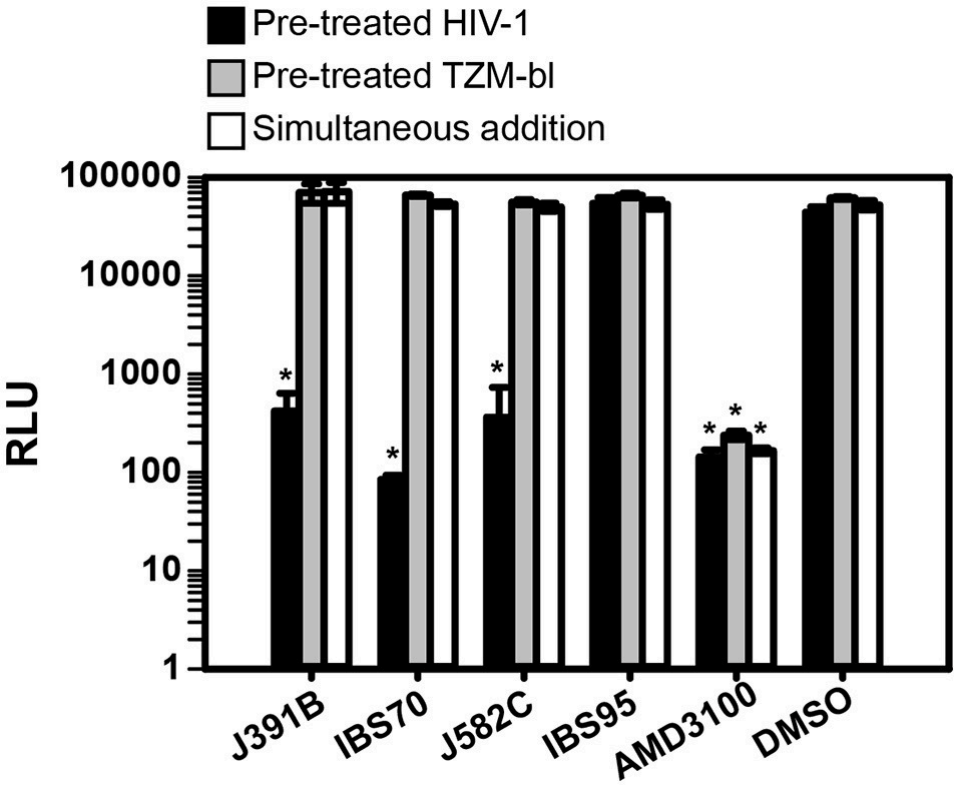
Lipidomimetics target the virion membrane. Lipidomimetic compounds were used to either pretreat HIV-1 (black bars) or TZM-bl reporter cells (gray bars), or compounds were added to TZM-bl reporter cells together with the virus (white bars). Concentrations used were 15 μM J391B, 10 μM IBS70; 82 μM J582C, 20 μM IBS95, 0.82% DMSO, and 0.5 μM AMD3100. TZM-bl reporter cells were harvested 42–45 h p.i. and luciferase activity induced by newly produced HIV-1 Tat was measured and is shown as relative light units (RLU). Data represent the mean ± SD of two replicate experiments with at least three replicas each; ^*^represents a significant (*p* < 0.01) decrease when compared to the DMSO control.

**Figure 5:**
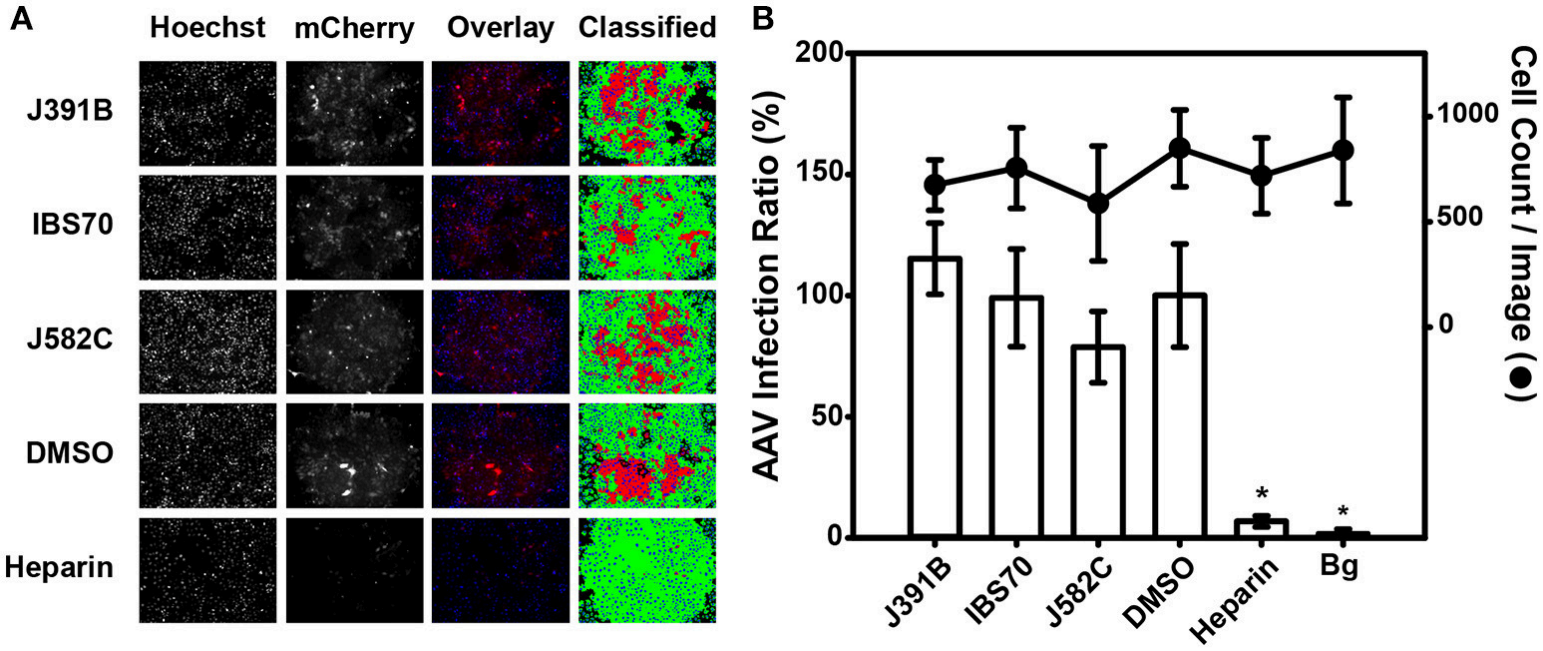
Lipidomimetics show no effect on non-enveloped AAV. Representative fluorescence images of TZM-bl cells infected with AAV expressing the reporter mCherry. AAV was pretreated with lipidomimetics at 12 μM J391B, 4 μM IBS70, or 40 μM J582C. Heparin (50 μg/ml), which is known to inhibit AAV entry, was used as positive control, IBS95 (20 μM) and DMSO (0.82%) as negative controls. Cell nuclei were stained by Hoechst 33342 (left panel) and AAV infection was recorded from mCherry fluorescence (second panel). The third panel shows an overlay of the two stains, and the right panel assignment of positive (red) and negative (green) cells is based on a proprietary algorithm. **(B)** Quantitation of automated fluorescence readout and cell counts. Fluorescent images as in **(A)** were automatically quantified for cell number (based on nuclear stain, right axis) and mCherry fluorescence (AAV infection ratio, left axis). Data represent the mean ± SD of two replicate experiments with sixteen replicas each; ^*^ represents a significant (*p* < 0.01) decrease when compared to the DMSO control.

To study the mechanism of inhibition, we first analyzed whether the lipidomimetic compounds acted on the virus or on the host cell. For this purpose, pretreatment of HIV-1 was compared with pretreatment of target cells prior to addition of virus, on the one hand, and to simultaneous addition of virus and compound on the other (Figure 4). All three active compounds reduced HIV-1 replication to background levels when virus was pretreated, whereas treating target cells prior to infection or simultaneously adding virus and compound had no effect on HIV-1 infectivity (Figure 4). To examine whether pretreatment of cells or simultaneous addition of compound and virus exhibited any antiviral effect, lipidomimetics were applied at 2–5-fold higher concentrations than required for effective pretreatment of the virus (cp. Figure 2). These concentrations were still well below the maximal tolerated concentrations in TZM cells (Figure 3). In contrast to lipidomimetics, AMD3100, which functions by blocking the CXCR4 co-receptor essential for HIV-1 entry, inhibited viral infection irrespective of the mode of addition. AMD3100 is a hydrophilic compound (logP = −0.34)^1^ that reaches the co-receptor from the aqueous phase, whereas the lipidomimetics with logP > 4 are quantitatively taken up by biological membranes and, where present, bind to lipoproteins and other hydrophobic molecules. Thus, the lipidomimetic compounds appeared to exert their effect by inactivating the virus and, therefore, had to be added prior to infection. To determine any potential non-specific effect on a non-enveloped virus, we also tested the lipidomimetic compounds against the parvovirus AAV, applying twice the effective concentrations required for HIV-1. Preincubation of AAV with any of the compounds did not affect infectivity, while heparin blocked AAV infection as expected, since it competes with AAV receptor engagement (50) (Figure 5). These results are consistent with an effect of the lipidomimetics on the HIV-1 membrane.

### Lipidomimetics inhibit HIV-1 entry at the fusion step

**Figure 6:**
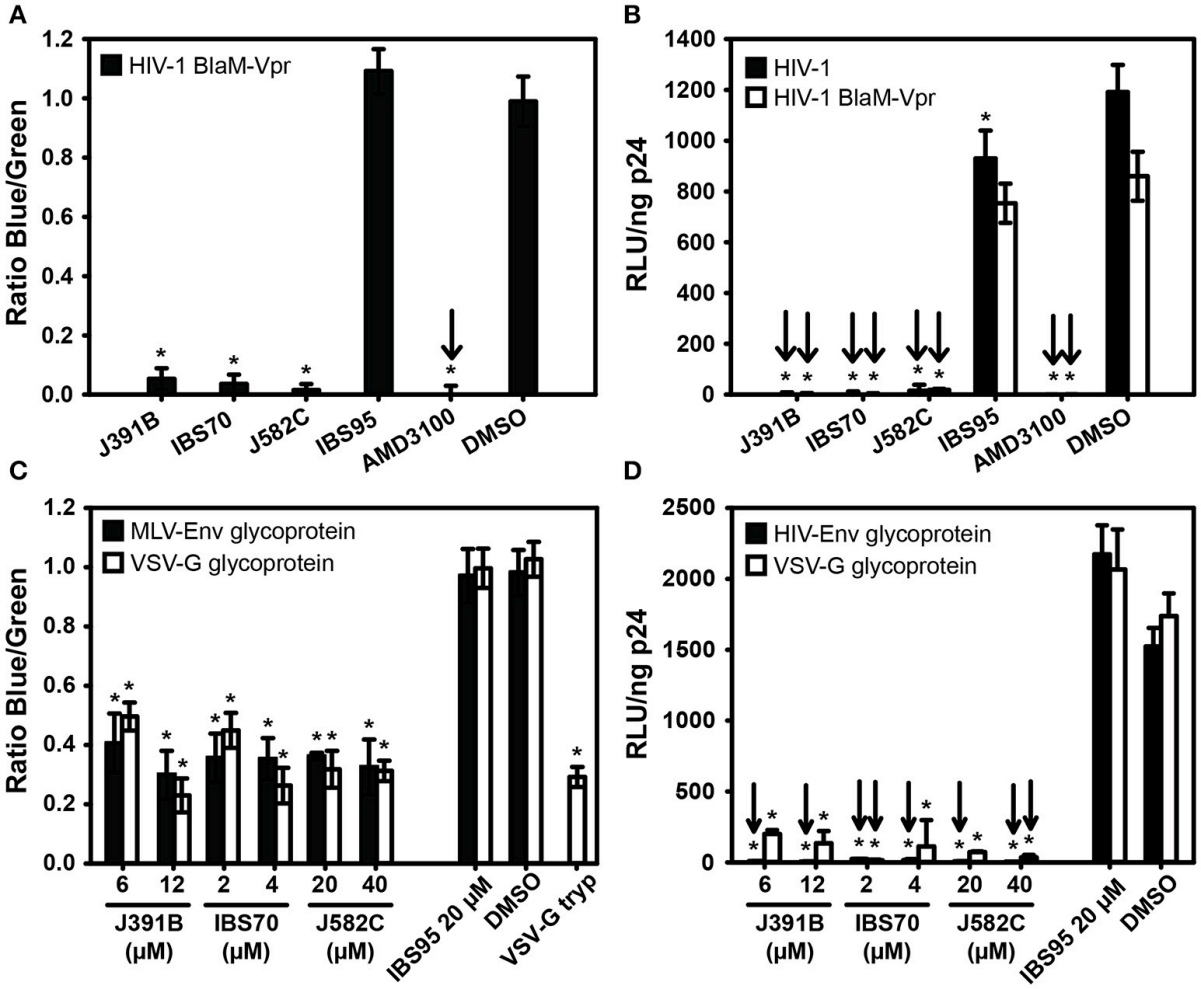
Mapping effects of lipidomimetics to HIV-1 entry. Cytoplasmic entry. HIV-1 carrying BlaM-Vpr was pretreated with 6 μM J391B, 2 μM IBS70, 20 μM J582C, 20 μM IBS95, 0.82% DMSO, or 0.5 μM AMD3100 and added to TZM cells as described in Figure 2. Cytoplasmic entry of HIV-1 was analyzed by determining the mean fluorescence for the cleaved (blue) and uncleaved (green) CCF2 substrate after 17 h. The graph shows the ratio of the blue and green fluorescence signal normalized against the DMSO control, which was set to 1. Data represent the mean ± SD of three replicate experiments with nine replicas each; ^*^ represents a significant (*p* < 0.01) decrease when compared to the DMSO control. **(B)** Comparison of BlaM-Vpr-carrying and unmodified HIV-1. HIV-1 with (white bars) or without (black bars) BlaM-Vpr was pretreated with compounds as in **(A)** and used to infect TZM-bl reporter cells. Relative infectivity is shown as RLU per ng of p24. Data represent the mean ± SD of two replicate experiments with six (white bars) and nine (black bars) replicas each; ^*^represents a significant (*p* < 0.01) decrease when compared to the DMSO control. **(C)** Influence of viral glycoproteins and entry route on sensitivity to compounds. HIV-1 was pseudotyped with the glycoproteins of Friend ecotropic MLV (black bars) or VSV (white bars). Pseudotyped viruses carrying BlaM-Vpr were pretreated with 6 and 12 μM J391B, 2 and 4 μM IBS70, 20 and 40 μM J582C or control compounds followed by infection of TZM-bl cells (for VSV-G pseudotypes) or DFJ8 cells (for MLV pseudotypes). Cytoplasmic entry was quantified by determining the ratio of blue and green fluorescence normalized against the DMSO control as in **(A)**. VSV-G pseudotyped particles treated with trypsin served as an entry non-competent control. Note that the signal for this control, which determines the background in this experiment, was similar to that of the compound-treated viruses. Data represent the mean ± SD of three replicate experiments with six replicas each; ^*^represents a significant (*p* < 0.01) decrease when compared to the DMSO control. **(D)** HIV-1 carrying its cognate glycoprotein (black bars) or pseudotyped with VSV-G (white bars) and containing BlaM-Vpr was pretreated with compounds as in **(C)** and used to infect TZM-bl cells. Relative infectivity is shown as RLU per ng of p24. Data are the mean ± SD of two replicate experiments with six replicas each; ^*^represents a significant (*p* < 0.01) decrease when compared to the DMSO control.

The mode-of-addition experiments suggested that lipidomimetics act by blocking viral entry through effects on the HIV-1 membrane. To directly address this question, we made use of a quantitative HIV-1 entry assay based on incorporation of a Vpr-β-lactamase fusion protein (Vpr-BlaM) into replication-competent HIV-1 particles during virus production (37). Once productive fusion with target cells occurs, β-lactamase is released from the virion into the cytoplasm, where it can cleave a fluorescent substrate, CCF2, loaded immediately after viral infection. Relative fusion activity is quantified as the fluorescence ratio of the cleaved and uncleaved fluorophores. HIV-1 particles pre-incubated with lipidomimetics exhibited a strong reduction of virus entry in this assay, which was also observed for AMD3100, but not for the control compound IBS95 (Figure 6A). To ensure that the infectivity of Vpr-BlaM viruses was comparable with wild-type HIV-1 and to correlate the effects on entry with inhibition of infection, an infection experiment was performed in parallel. No significant difference in infectivity was observed between wild-type HIV-1 and Vpr-BlaM virus, and both viruses were equally affected by lipidomimetics (Figure 6B). Thus, inhibition of HIV-1 infection by lipidomimetics maps to the viral entry step.

To determine whether the effect of the lipidomimetic compounds is dependent on the HIV-1 glycoproteins or the viral entry route, we performed experiments on HIV-1 pseudotyped with other viral glycoproteins. Pseudotyping is achieved by producing virus particles lacking their cognate viral glycoproteins, but randomly incorporating heterologous glycoproteins synthesized by co-transfecting the same producer cell with an expression vector for the glycoprotein of another virus. Pseudotyping with e.g., the glycoprotein of vesicular stomatitis virus (VSV-G) also changes the viral entry route as VSV enters target cells through a pH-dependent endosomal route (51). In contrast, pseudotyping with Friend ecotropic MLV glycoproteins yields viral fusion and entry at the plasma membrane in a pH-independent manner (52). We therefore produced HIV-1 Vpr-BlaM particles carrying the glycoproteins of either VSV or MLV and tested their capacity to enter target cells after pretreatment with lipidomimetic or control compounds. As background controls, pseudotyped HIV-1 particles were trypsin-treated prior to inoculation. Viral infectivity was determined in parallel for pseudotyped HIV-1 particles treated with lipidomimetic and control compounds as described above. Similar to HIV-1 carrying its cognate envelope proteins, treatment with lipidomimetics efficiently and specifically inhibited cell entry by particles pseudotyped either with VSV or with MLV glycoproteins (Figure 6C). The effect on entry again correlated with that on virus infection (Figure 6D). Accordingly, the antiviral activity of the lipidomimetic compounds is not dependent on a specific envelope glycoprotein or on a particular entry pathway.

**Figure 7:**
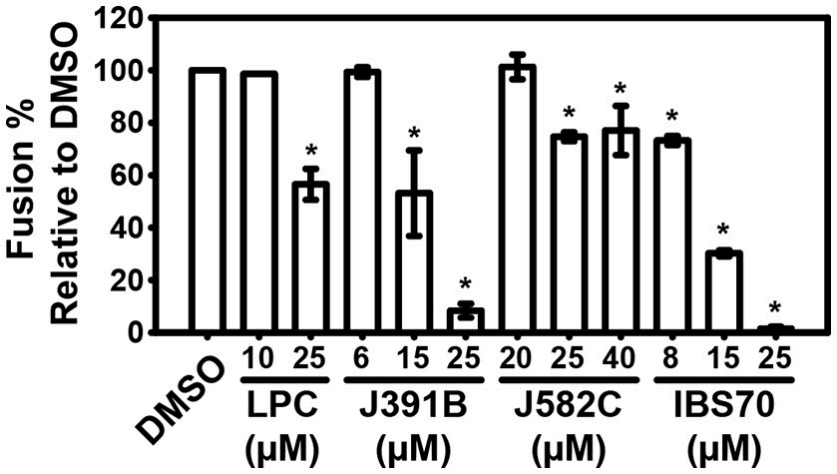
Inhibition of fusion by lipidomimetics. LUVs were composed of POPC:POPS:SM:Chol (18:15%:33:33%) to emulate viral particle membranes. Experiments were performed at 25°C with 0.1% FCS in 150 mM NaCl, 10 mM Hepes pH 7.4 buffer, 10 μM NBD/Rho LUV in 1 ml, incubated for 15 min with compounds or DMSO control (0.82%). Next, 40 μM of unlabeled LUV was added for 20 min. The stable background signal establishes 0% fusion. The fusion reaction was started by adding 0.5 μM MPER fusion peptide. Membrane fusion was measured as the increase of N-NBD-PE fluorescence due to intervesicular lipid mixing and consequent dilution of the fluorophore (NBD) and quencher (Rho). In the absence of MPER there was no fluorescence increase (0% fusion). Finally, TX-100 (0.1%) was added to establish the 100% fusion signal. Data are the mean ± SD of two replicate experiments with three replicas each. ^*^represents a significant (*p* < 0.01) decrease when compared to the DMSO control.

To further study the mechanism by which lipidomimetics inhibit viral entry, we made use of a liposome-based fusion inhibition assay (44). For this purpose a fusogenic peptide from HIV-1, the gp41 MPER and synthetic LUVs were used (18, 53–55). As a suitable match for the lipidomimetics, LPC was chosen as the positive control compound. LPC is a well-known fusion inhibitor lipid (19) with inverted cone-shaped structure, which affects membrane curvature and therefore inhibits liposomal lipid mixing. Concentration-dependent inhibition of MPER-induced fusion by lipidomimetics was studied (Figure 7). The extent of virus and liposome lipid mixing correlated with the compounds' inhibition of virus infectivity (IC_50_). IBS70 inhibited lipid mixing most effectively, followed by J391B and J582C. Thus, lipidomimetics were capable of inhibiting viral entry at the membrane fusion step.

### Effects of lipidomimetics on HIV-1 stability and virion density

**Figure 8:**
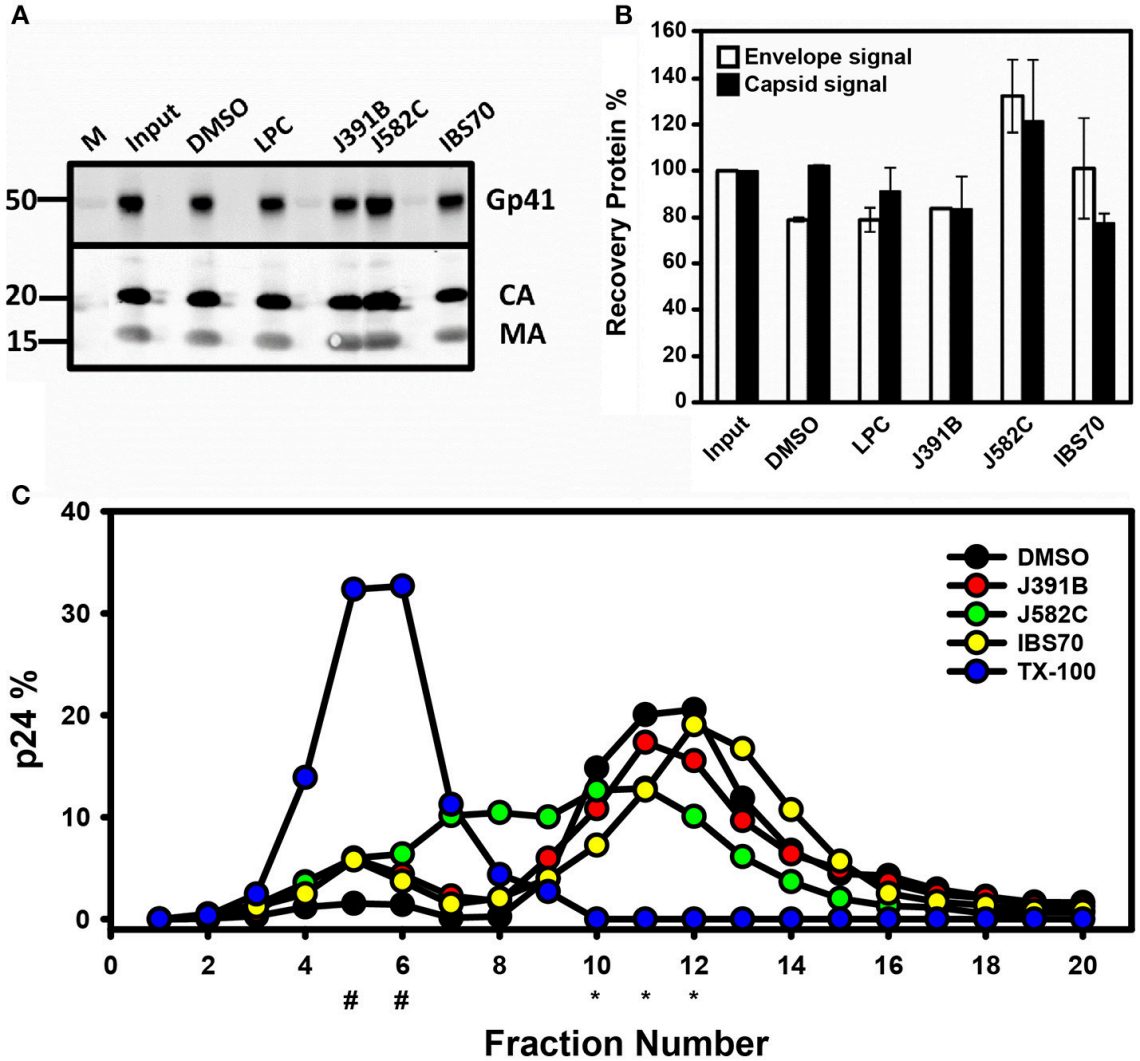
Influence of lipidomimetics on particle stability and density. **(A)** Virus stability. Purified non-infectious HIV-like particles derived by transfection of pCHIV (3 μg of CA) were treated with 6 μM J391B, 8 μM IBS70, 20 μM J582C, 2.5 μM LPC, or DMSO (0.35%) for 30 min at 37°C. Subsequently, particles were recovered by ultracentrifugation and analyzed by Western blot using antisera against the HIV-1 trans-membrane glycoprotein gp41 (ENV) (41 kDa), CA (24 kDa), and MA (17 kDa). Input signal refers to control virus before treatment. **(B)** Virus stability. Virus treated and recovered as in **(A)** was analyzed by quantitative Western blot. Measured in LiCoR quantitative system. **(C)** Virus buoyant density. Non-infectious HIV-like particles derived by transfection of pCHIV (3 μg of CA) were treated with 6 μM J391B, 8 μM IBS70, 20 μM J582C, 0.5% TX-100, or DMSO (0.35%) as in **(A)** and subsequently subjected to equilibrium density gradient centrifugation. Gradient fractions were collected from the top and virus amounts were quantified by Western blot. Data represent the mean of two replicate experiments with five replicas each, # indicates sucrose density fractions where soluble p24 was expected (1.07 g/cm^3^) and ^*^the fractions where intact virus was expected (1.17–1.2 g/cm^3^).

Detergent-like microbicides, e.g., nonoxynol, disrupt membranes non-specifically, thus inactivating HIV-1 with concomitant toxicity (56). To investigate whether virus disruption plays a role in the antiviral activity of the lipidomimetic compounds, purified non-infectious HIV-1-like particles and infectious HIV-1 were treated with compounds at approximately the IC_90_ and subsequently recovered by ultracentrifugation. Western blots detecting capsid, matrix, and envelope proteins and their quantifications revealed no effect of compound treatment on virus recovery and protein composition (Figures 8A,B and Figure S2).

Previous studies demonstrated shifts in virion density when viral membrane composition was altered by changes in cholesterol concentration or its replacement by cholesterol analogs (13). In order to monitor potential density shifts following treatment with lipidomimetics, both non-infectious HIV-like particles (Figure 8C) and infectious HIV-1 (Figure S2) were compound-treated and analyzed by equilibrium centrifugation on continuous sucrose density gradients. HIV-1 protein distribution in gradient fractions was subsequently assayed by quantitative p24 Western blots or ELISA. Purified solvent-treated HIV-1 served as reference with the virus peak in fractions 10–13 at a density ranging from 1.16 to 1.2 g/cm^3^ (Figure S3). In contrast, virus treatment with detergent Triton X-100 led to a complete loss of the virus peak and recovery of soluble p24 antigen in the top fractions of the gradient (Figure 8C and Figure S2). Treatment of viral particles with J391B did not influence virus density and resulted in a similar virus yield detected in fractions 10–13 as observed for solvent-treated virus. However, IBS70 elicited a minor increase in virus particle density. As opposed to the other lipidomimetics, a reduction in density to 1.12–1.14 g/cm^3^ occurred upon exposure of HIV-1 to J582C, a sphingosine mimic (Figure 8C and Figure S2). Effects on particle density may be due to insertion of the lipidomimetic into the viral membrane. However, changes in virion density were not proportional to antiviral activity. Only a small amount of soluble p24 was released by lipidomimetic-treated virus (Figure 8C and Figure S2), confirming that the compounds did not act by affecting virion stability.

### Effect of lipidomimetics on HIV-1 membrane order

Our previous studies had shown that the fluorescent dye laurdan can be used to determine the degree of membrane order in virus particles (9), and we therefore applied laurdan staining to detect potential changes in membrane order upon treatment of HIV-1 with lipidomimetics. Laurdan is homogeneously distributed within the membrane and has an emission maximum around 490 nm for fluid (liquid disordered, l_d_) membranes and around 440 nm for condensed membranes (l_o_, and gel phase or solid ordered, S_o_). The phase state of a membrane can thus be quantified by its GP value, which is defined as the normalized intensity ratio of the two emission channels and provides a relative measure of lipid order (9, 57, 58). GP values between 0.25 and 0.5 indicate l_o_ structure at the respective temperature, while GP values < 0.25 indicate liquid-disordered (l_d_) structure (59).

**Figure 9:**
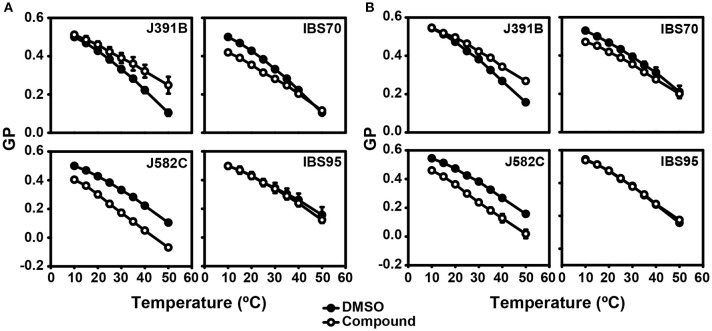
Effect of lipidomimetics on HIV-1 membrane order. **(A)** Comparison of temperature-dependent GP profiles of HIV-1 with (◦) or without (•) lipidomimetic treatment. Viruses were stained with 5 μM laurdan for 20 min, treated with 6 μM J391B, 8 μM IBS70, 20 μM J582C, 7 μM IBS95, or DMSO (0.35%) for 30 min at 37°C, and analyzed as described in experimental procedures. GP values were calculated from emission spectra recorded at each temperature. Data are the mean ± SD of three replicate experiments.**(B)** Comparison of temperature-dependent GP profiles of LUV produced from extracted viral lipids with (◦) or without (•) compound treatment. Conditions of treatment and analysis were as in **(A)**. Data are the mean ± SD of three replicate experiments.

Prior to compound treatment and laurdan staining HIV-1 was inactivated with AT-2, which covalently modifies the essential zinc fingers in the viral nucleocapsid protein (30) without altering the membrane (9). Inactivated virus was treated or not with lipidomimetics or control compounds and subsequently labeled with laurdan. Particles were recovered by ultracentrifugation, re-suspended in buffer and subjected to fluorescence spectroscopy. Control experiments excluded a direct interaction of lipidomimetics with laurdan (Figure S4). Emission spectra were recorded for treated and untreated viruses at different temperatures and the corresponding GP values were calculated. The charts of GP as a function of temperature of solvent-treated particles (Figure 9, filled circles) exhibited similar shapes and values as previously observed (9). No transition temperature inflection was visible, as expected for membranes with high cholesterol content (60). Upon treatment with J391B, J582C, and IBS70, HIV-1 particles exhibited a change in GP pattern while treatment with the inactive compound IBS95 was indistinguishable from the solvent control, which was only subject to temperature-induced order decrease (Figure 9A). The cholesterol analogue J391B increased GP values of treated HIV-1 particles, generating greater membrane rigidity and counteracting the effect of temperature increase. Conversely, treatment with IBS70 and, most severely, the sphingosine-analogue J582C induced a decrease in GP values, reflecting enhanced membrane fluidity in the l_d_ range (GP < 0.25). While the reduction of membrane order by IBS70 was abated with increasing temperature, the disordering activity of J582C was constant and additive to the temperature effect (Figure 9A).

The proteins resident in biological membranes enforce an asymmetric distribution of lipids between the two leaflets, whereas liposomes lacking proteins exhibit a nearly symmetric lipid distribution. To determine whether the effect of the compounds on viral membrane structure is exclusively mediated by viral lipid composition, GP values were determined for LUV composed of HIV-1-derived lipids. To this end, lipids were extracted from purified HIV-1 particles and used to prepare LUV with a diameter of ~100 nm (43). These HIV-derived LUV have the lipid composition of HIV-1 but, according to their physicochemical characteristics, possess a random and presumably symmetric distribution of lipids. HIV-1 lipids-derived LUV were treated or not with the different compounds, labeled with laurdan, and GP was recorded as a function of temperature (Figure 9B). Similar to the results observed for wild-type HIV-1, the control compound IBS95 did not alter LUV structure; IBS70 and J582C caused a temperature-dependent or independent decrease in membrane order, while J391B stabilized membrane order against rising temperature (Figure 9B). The modifications induced by all compounds in LUV were very similar to the ones observed for the complete virus, indicating that their effects were independent of viral or cellular proteins and did not require membrane asymmetry.

### Phosphatidylserine-specific enhancement of membrane order by steroidal amine J391B

**Figure 10:**
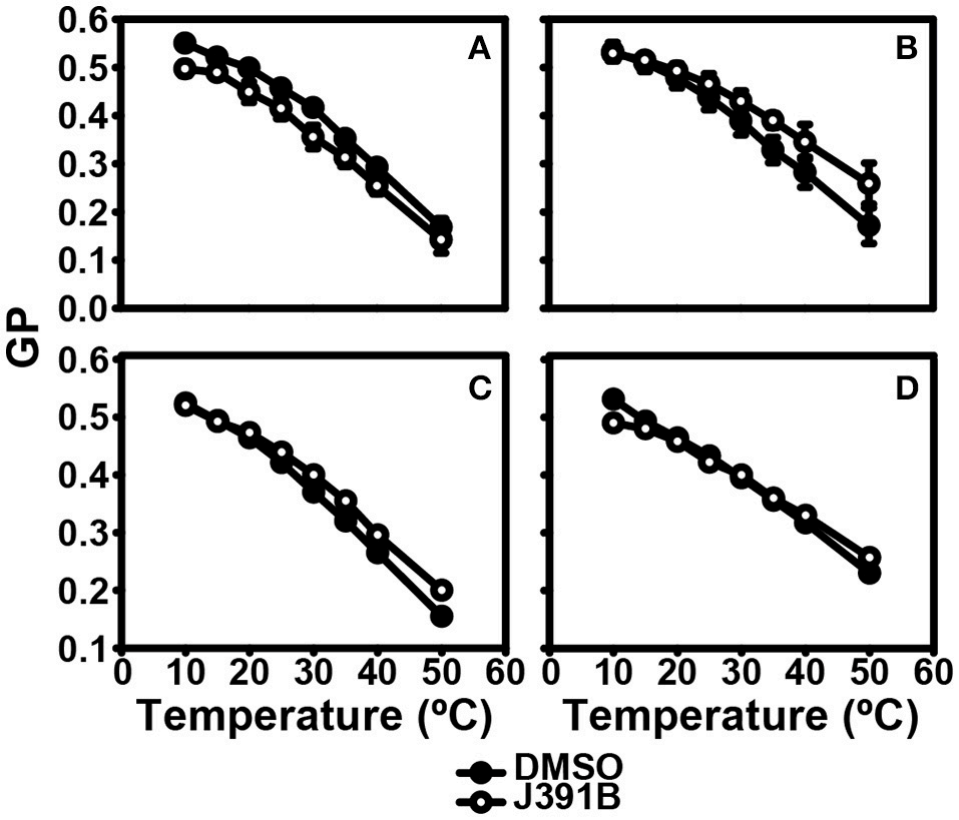
Lipid species requirement for J391B membrane activity. Comparison of temperature-dependent GP profiles of LUVs composed of: **(A)** POPC:DPPC:SM:CHOL (25:16:14:45%). **(B)** POPC:DOPS:DPPC:SM:CHOL (10:15:16:14:45%). **(C)** POPC:DOPG:DPPC:SM:CHOL (10:15:16:14:45%). **(D)** POPC:DOPA:DPPC:SM:CHOL (10:15:16:14:45%) with (◦) or without (•) treatment with 6 μM J391B for 30 h at 37°C. Data represent the mean ± SD of three replicate experiments.

**Figure 11:**
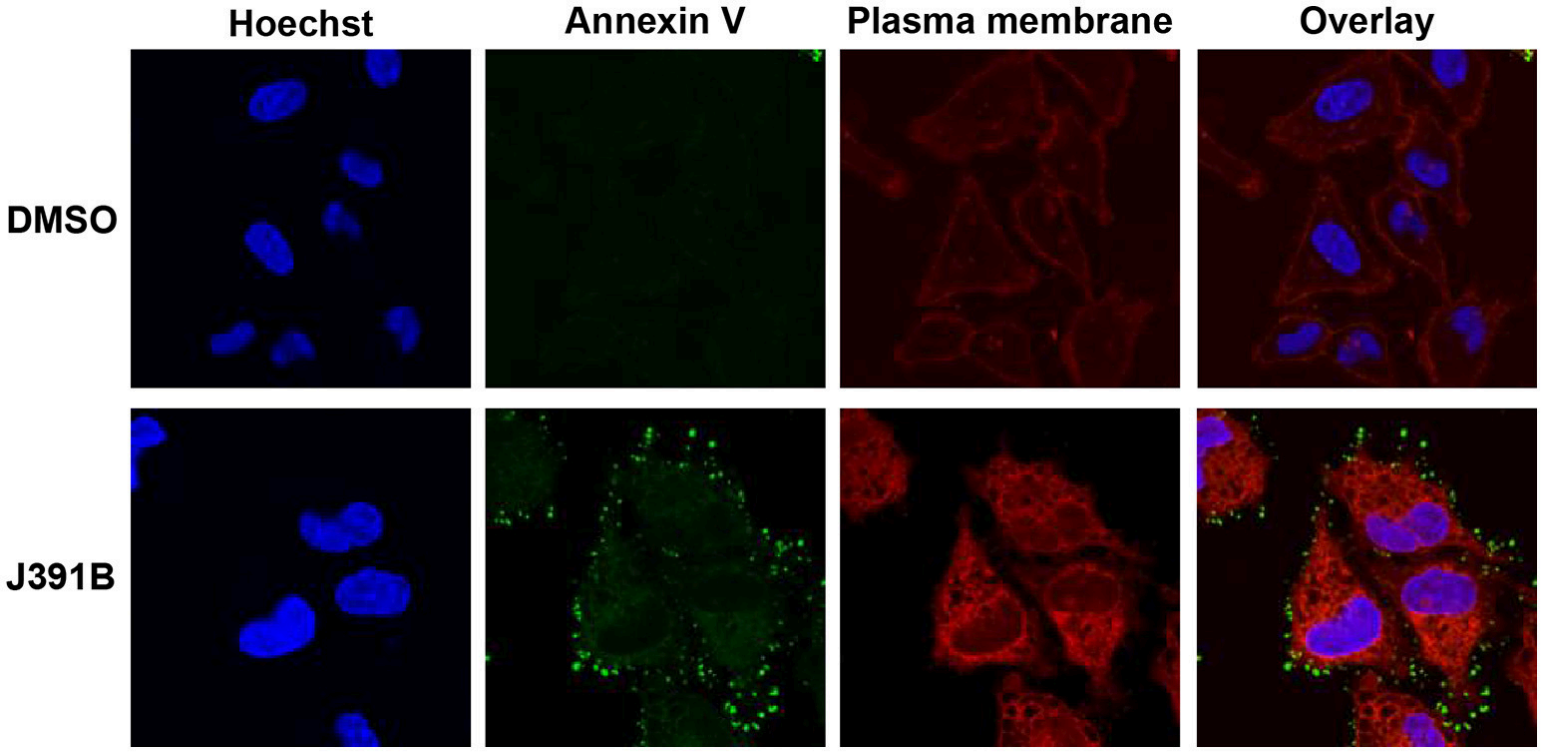
J391B binding to the cell membrane triggers exposure of PS in the outer leaflet. HeLa cells were treated with 10 μM J391B or with 0.82% DMSO. The plasma membrane was labeled with Wheat Germ Agglutinin (red); exposed PS was detected by annexin V (green) and nuclei were stained with Hoechst 33342 (blue). The figure is a representative image of two replicate experiments.

Further studies with LUVs of different membrane composition revealed an interesting lipid headgroup requirement for the effect of J391B on membrane order. While this compound enhanced membrane order in HIV-1 particles and LUV reconstituted from the complete set of HIV-1 lipids (Figures 9A,B), a slight decrease of membrane order was observed upon J391B treatment of LUV consisting only of cholesterol, sphingomyelin and phosphatidylcholine (the most abundant HIV-1 lipids; Figure 10A). Since J391B is positively charged at neutral pH (Figure 1), apparently the availability of negatively charged lipid head groups determined a switch between J391B reducing, maintaining or increasing membrane order. The HIV-1 lipidome exhibits an enrichment of phosphatidyl serine (PS) compared with the plasma membrane of producer cells (8). LUV composed of the same quaternary mixture as above but including PS substituting for part of the phosphatidyl choline exhibited increased membrane rigidity upon J391B treatment, although the amount of saturated fatty acids had been decreased 2.5 times as a result of the higher level of unsaturated fatty acids in PS (Figure 10B). The effect of the compound resembled that on LUV consisting of HIV-1 extracted lipids or on complete virus (Figure 9). Replacing PS with other negatively charged lipids, phosphatidyl glycerol or phosphatidic acid, which hardly occur in the HIV envelope, yielded little or no effect on membrane order following J391B treatment (Figures 10C,D), indicative of a specific molecular interaction of J391B with PS rather than mere charge neutralization. To explore this molecular interaction in an independent approach HeLa cells were treated with J391B for 30 min and stained with PS-specific annexin V. J391B treatment elicited exposure of PS on the outer leaflet of the plasma membrane (Figure 11) whereas the DMSO-treated control gave no signal. This observation indicates that J391B interaction with cell membranes induced PS externalization without apparent signs of apoptosis.

### J391B and J582C exhibit synergistic effects on HIV-1 infectivity

**Figure 12:**
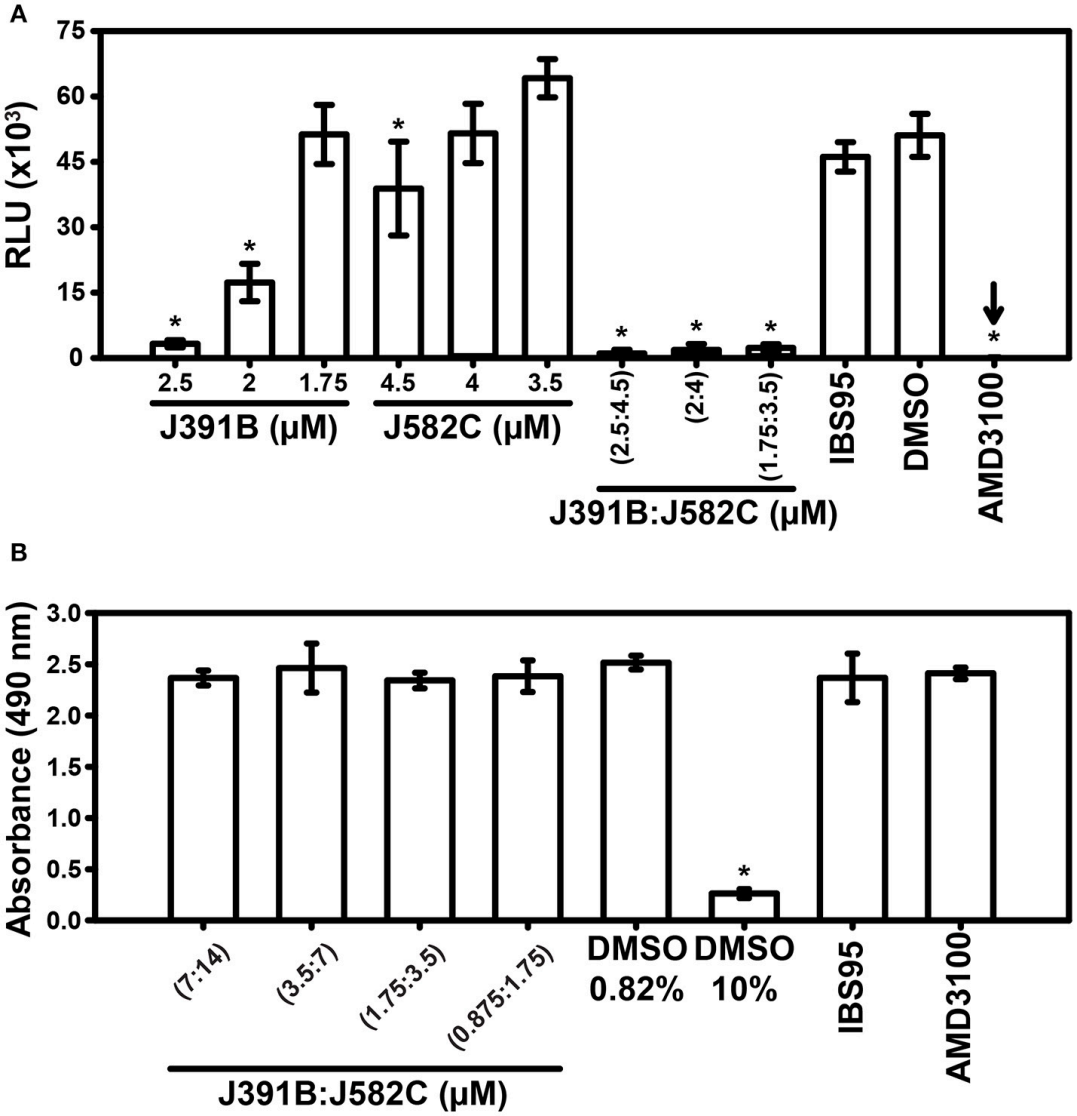
Effect of mixtures of J391B and J582C on HIV-1 infectivity and cytotoxicity. **(A)** HIV-1 was treated with compounds at the indicated concentrations as described in Figure 2 followed by infection of TZM-bl cells and luciferase assay after 42 h. Data represent the mean ± SD of three replicate experiments with nine replicas each; ^*^ represent a significant decrease (*p* < 0.01) when compared to DMSO. **(B)** MTT cytotoxicity assay for treatment with compound mixtures. The same J391B: J582C (1:2) molar ratio as in **(A)** at expanded concentration range was tested. DMSO (0.82%), IBS95 (7 μM) and AMD3100 (0.5 μM) served as controls and 10% DMSO as toxicity control. Data are the mean ± SD of three replicate experiments with four replicas each; ^*^represent a significant decrease (*P* < 0.01) when compared to the 0.82% DMSO control.

Given that J391B and J582C induced opposing effects on membrane structure, we next asked whether they would antagonize each other. To study their combined effects, the two compounds were mixed at different concentrations, and the infectivity of HIV-1 treated with either compound alone or with the various mixtures was analyzed using a luciferase reporter assay (Figure 12A). Again, AMD3100 was employed as the positive and IBS95 as the negative control compound. Titration experiments showed that J391B had no effect on infectivity at a concentration of 1.75 μM and J582C was inactive at 3.5–4 μM (Figure 12A). Yet a mixture of both compounds at concentrations where either compound alone was inactive strongly inhibited viral infectivity. Thus, J391B and J582C acted synergistically. Enhanced inhibition was not due to toxicity as shown by the parallel MTT test (Figure 12B).

**Figure 13:**
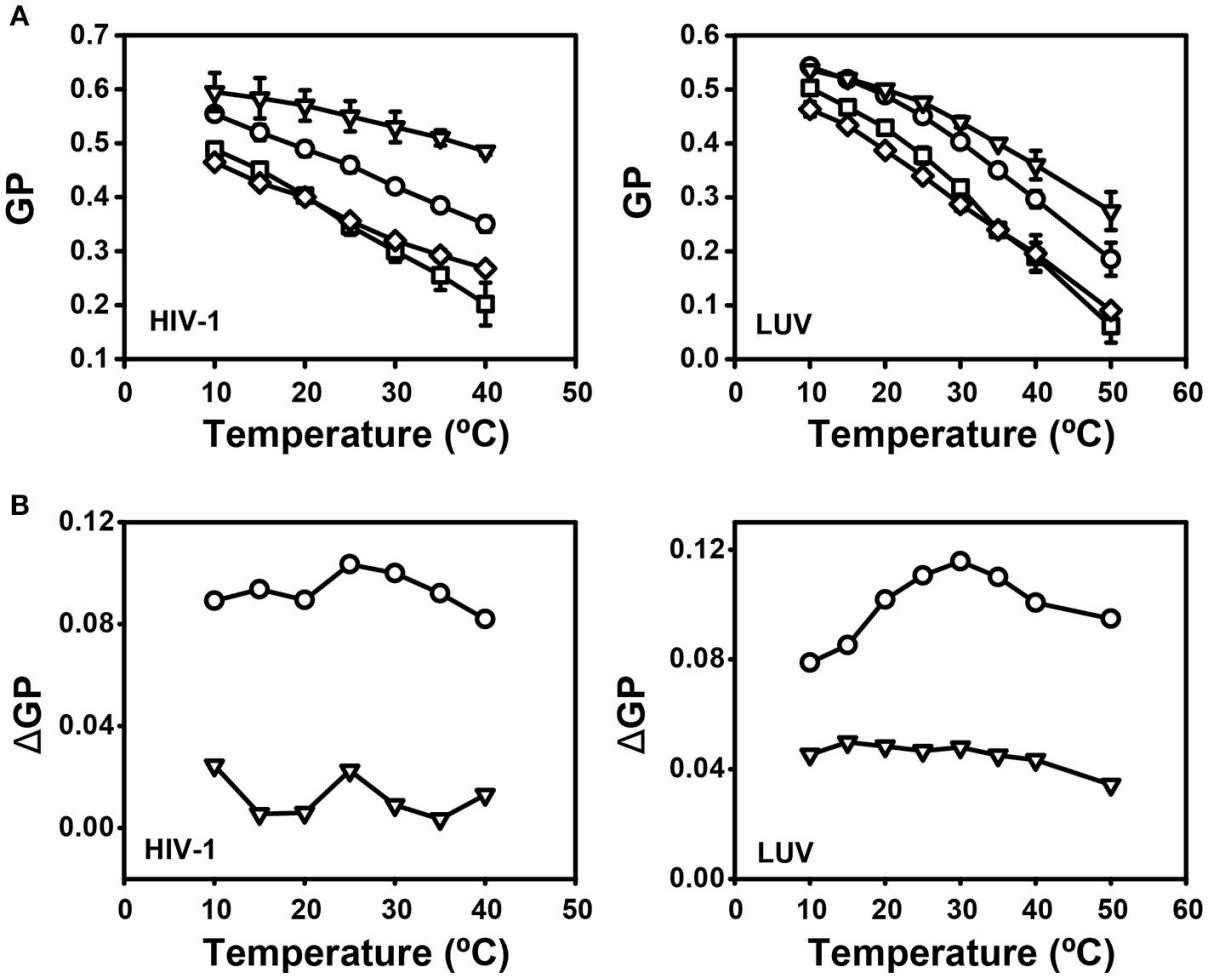
Effect of mixtures of J391B and J582C on HIV-1 membrane structure. **(A)** Comparison of temperature-dependent GP profiles of HIV-1 (left panel) and LUV composed of HIV-1 lipids (right panel). Virions or LUV were treated with 0.82% DMSO (circles), 7.5 μM J391B (triangles), 15 μM J582C (squares) or the mixture of 7.5 μM J391B and 15 μM J582C (diamonds). Data represent the mean ± SD of three replicate experiments. **(B)** Temperature-dependent GP profiles for HIV-1 NL4-3 (left panel) or LUV (right panel) treated with compound mixtures or individual compounds were plotted against DMSO-treated controls. Data are from the experiment shown in **(A)**. In order to address whether the effect of the compound mixture is additive or synergistic, we plotted the GP profile for particles treated with the mixture of J391B and J582C relative to DMSO controls (circles) or against the extrapolated additive plot for particles separately treated with both compounds (triangles).

We next examined whether the synergistic effect on viral infectivity corresponded to detectable alterations in HIV-1 membrane order. HIV-1 particles and LUV composed of HIV-1 lipids were treated with individual or mixed compounds and GP profiles were recorded (Figure 13A). Interestingly, the compound mixture delivered a GP pattern close to that of J582C but with a temperature profile more like the one of J391B. The complex outcome at the level of membrane order was neither an antagonistic nor an additive effect (Figure 13B).

## Discussion

Targeting the lipid membrane of enveloped viruses is an attractive approach for the development of antivirals applied either systemically or on mucosal surfaces. Detergent-based structures have been developed as topical microbicides (56), but shown to cause toxicity problems upon *in vivo* application (61, 62). Alternative approaches attempted the extraction of key lipids like cholesterol with cyclodextrin derivatives (12) or promoted alteration of membrane fusion properties by e.g., inserting inverted cone-shaped non-lipidic compounds into the viral membrane (20). Here, we screened a library of raft lipid-like lipidomimetics as potential antiviral agents against HIV-1 and identified several compounds, which inhibited viral infection at the entry stage and induced structural alterations in the viral membrane.

A qualitative structure-activity analysis of antiviral vs. inactive compounds from the screen yielded the following results: the hydrophobic anchor of active sterol-like molecules often included a single oxygen atom, attenuating the hydrophobicity of the sterol-like scaffold. There was no preference for the α- or ß-sterol configuration. The hydrophobic anchor of active aliphatic lipidomimetics was a single aliphatic chain ≥ 14–18 C-atoms. Compounds with more than one aliphatic chain were inactive due to their poor diffusibility, despite the presence of serum lipoproteins. Among the inactive compounds, we selected IBS95, which is similar to the generically active structures but has two ring moieties in its head group.

Head groups of the active compounds often encompassed OH-groups and/or a single aromatic or heterocyclic ring, and presented at least one nitrogen atom, usually positively charged or available for protonation. These observations agree with the findings of (63) who showed that cholesterol derivatives with positively charged head groups disrupt or augment membrane order and, in both cases, interfere with influenza virus infection. It is not unexpected that any modulation of the initial, optimal viral membrane order impairs infectivity, since all physiological processes exhibit concentration and temperature optima, often in the context of protein function. Likewise, biological membranes possess a distinct order determined by lipid species concentrations and temperature, essential for their functions (64). Specifically composed lateral membrane phases underly organellar and viral membrane dynamics (fusion, budding, and fission) and trafficking (65).

Lipidomimetics inhibited HIV-1 infectivity in a concentration-dependent manner with IC_50_ values in the low micromolar range. Activity required prior interaction with the viral membrane, while no effect was seen upon pre-incubation of targets cells with the compounds. Given that these compounds are likely to bind to both virus and cell membranes, this suggests that their interaction with cellular membranes may not influence viral entry or/and that compounds are rapidly removed from cellular but not from viral membranes. Indeed, ATP-dependent transporters (ABC transporters, P glycoprotein) remove foreign amphiphilic compounds from cell membranes (66), and may also work in this manner on the compounds studied here. Further experiments confirmed that the viral membrane is indeed the target: Lipidomimetics inhibited HIV-1 entry independent of the viral envelope glycoprotein and the specific entry pathway, while no effect was observed for the non-enveloped virus AAV. We conclude that the described lipidomimetics directly target the viral membrane and alter its capacity to fuse with the host cell membrane. This effect suggests potential against a wider spectrum of enveloped viruses, while the lipid-dependent activity differences (see below) may restrict activity to membranes with a defined lipid composition.

Studies of virus stability showed that the compounds did not disrupt particle integrity although J582C caused a shift in buoyant density of the viral particle. Sphingosine-like (J582C) and other long acyl-chain compounds (as IBS70 or IBS95) contain a single hydrocarbon chain and have inverted cone-shape geometry like LPC, generic structures that induce positive curvature and thus inhibit membrane fusion (19). While LPC itself is toxic, less toxic compounds have been shown to inhibit virus entry due to their inverted cone-shape structure (20). These RAFIs consist of a nucleoside coupled to perylene and have a wide antiviral spectrum against enveloped but not naked viruses. A similar broad antiviral spectrum is exhibited by the amphiphilic fusion inhibitor, aryl methylene rhodanine derivative LJ001 (48). Both LJ001 and RAFIs were shown to target membranes though they do not structurally resemble lipids, but the effect of LJ001 is actually mediated by photosensitization (67), unrelated to raft modulation. More relevant, cosalane is a cholestane derivative with an oversized headgroup comprising disalicylmethane with activity against enveloped viruses (68). The cholestane moiety of cosalane is reported to insert into the cell membrane and/or the viral envelope, from where the large disalicylmethane moiety protrudes and blocks the interaction between gp120 and CD4 (69). For the activity of cosalane the membrane raft-targeting property of the lipid anchor (cholestane) appears to be important, however, the mode of action does not involve the disruption of membrane raft domains of the virus envelope. This compound would be defined as a raftophile; it is probably not a raft modulator or disrafter (45, 63).

A number of lipidic HIV inhibitors were previously studied with regard to modifying both the host cell and viral membrane, their fluidity and lipid domain structure [reviewed by (70)]. The natural compounds glycyrrhizin and fattiviracin FV-8 possess large, neutral, hydrophilic headgroups, and are structurally far removed from natural raft lipids from which our lipidomimetics originated. In a review of the potential relevance of membrane raft targeting by natural products as an anti-HIV strategy (71), a considerable body of literature on betulinic acid derivatives is cited, structurally related to the lupene derivative J391B, however, none of them carrying a 3-amino group that proved decisive for the specific HIV envelope-stabilizing activity of J391B described here.

In order to study the potential effect of lipidomimetics on membrane structure, we made use of laurdan staining, which allows rapid determination of differences in viral membrane order (9). Diametrically opposed alterations in membrane order were observed for the cholesterol analogue J391B, which increased membrane rigidity and counteracted temperature-induced melting, and the sphingosine analogue J582C, which increased membrane fluidity independent of temperature. IBS70 also increased the fluidity of the virus envelope, but its effect disappeared at higher temperatures. Membrane rigidification by the steroidal amine J391B only superficially resembled the effect of increasing the proportion of membrane cholesterol, the lipid fundamental to the existence of l_o_ phases (72, 73). Interestingly and unlike cholesterol, the effect of J391B on membrane order required the presence of PS. This lipid dependence was not caused by an unspecific electrostatic effect as substitution of PS by either phosphatidyl glycerol or phosphatidic acid at a similar concentration in the presence of J391B had little or no effect on membrane order. Surprisingly, introduction of J391B into membranes completely lacking PS and other negatively charged lipid headgroups increased membrane fluidity, similar to J582C and IBS70. We therefore hypothesize that there is a specific electrostatic interaction of J391B with PS, and this may create more rigid membrane structures. Annexin V staining of cells treated with J391B revealed a rapid exposure of PS on the cell surface without detectable signs of apoptosis (Figure 11), indicating that PS flipping from the inner to the outer leaflet is trapped by the compound, which suggests a high binding affinity to PS. The viral membrane is highly enriched in PS compared to the producer cell plasma membrane (8), and this may make HIV-1 a particularly good target for J391B. As a low-molecular weight, membrane-inserting PS ligand, J391B is novel and structurally unrelated to the three hitherto described (non-lipidomimetic) PS binders (74).

Intriguingly, upon HIV engagement of its receptors, flipping of PS to the outer leaflet of the host cell target membrane has been reported to be important for HIV fusion. Zaitseva et al. (75) showed that HIV binding and formation of the pre-fusion Env-CD4-coreceptor complex leads to surface expression of PS. First, the complex triggers a Ca^2+^ signal, which in turn activates lipid scramblase TMEM16F that externalizes PS, which is then essential for the next step of fusion, gp41 restructuring and hemifusion. It will be interesting to determine if virion PS is also important for fusion and whether its function is inhibited by bound J391B. PS in the outer leaflet of the viral membrane has already been shown to be important for cell entry of other enveloped viruses (76), underlining the relevance of testing J391B activity against these viruses in the future.

Treatment of HIV-1 with the sphingosine-like compound J582C led to decreased membrane order and a concomitant decrease in virus density. Laurdan directly senses the abundance of water molecules within the membrane, which inversely correlates with membrane order (42). Conceivably, insertion of J582C into the viral envelope may cause membrane swelling by allowing more water to penetrate. Indeed, the bulkier, uncharged head group of J582C comprises three hydrogen-bond acceptors, as opposed to the small positively charged head group of sphingosine with its three hydrogen-bond donors (Figure 1). Natural sphingosine has a completely different behavior compared to the uncharged compound J582C. The positive charge of sphingosine appears crucial for its membrane activity [reviewed in (77)]. Sphingosine rigidifies the bilayer lipid acyl chains, as a result membrane permeabilization can occur due to the coexistence of domains of different fluidities (77, 78). On the other hand, J582C-induced positive membrane curvature in combination with water incorporation would tend to swell the membrane, explaining the observed decrease in particle density.

Based on the observed opposing effects of J391B and J582C on HIV-1 membrane order, experiments were performed with a mixture of both to investigate their potential antagonism. Counterintuitively, J391B and J582C synergistically inhibited HIV-1 infectivity, associated with an increased membrane fluidity apparently dominated by J582C, combined with a flatter temperature-dependent GP profile reminiscent of J391B alone. Dominant membrane order enhancement by the specific interaction between J391B and PS appears to be abrogated in the presence of J582C, yet the mixture of both distinct raft modulators appears to create a greater obstacle to fusion than each compound individually. Thus, in addition to the impact on global viral membrane order, as reported by the laurdan assay, membrane lipid mechanics at a smaller scale (as required for fusion) are a target of lipidomimetics, which seem to act as molecular “spanners in the works” of fusion. Further studies will be required to identify the precise mechanism of this synergistic effect.

We are aware that efforts toward selective drug delivery are the precondition to optimizing anti-HIV lipidomimetics, since their hydrophobicity facilitates indiscriminate absorption by cell membranes, followed by either uptake into the cell or expulsion via transporters and re-loading onto lipoproteins (66). Selective drug delivery may, for example, target natural, highly specialized HIV infection pathways. Appropriately engineered lipidomimetic-loaded ganglioside-containing vesicles may be a promising approach of interfering with primary mucosal infection. After targeting siglec-1-expressing mature dendritic cells, which are not productively HIV-infected, the vesicles would inactivate HIV encountered in the same intracellular sac-like compartment (79, 80).

Very little is known about the role of membrane order and fluidity regarding virus pathogenicity and how to modulate the physicochemical properties of the virus envelope to achieve a desired inhibitory phenotype. Studying lipid-modulating compounds like the ones described here provides a glimpse of this fascinating subject and may pave the way for future studies.

## Author contributions

BG, CS, and ML performed experimental infectivity and entry capacity data acquisition and analysis; JN-G and ML did lipid mixing assays, particle stability and density measurements; ML conducted laurdan experiments and cytotoxicity assays and identified J391B lipid requirement as well as the effect of compound mixtures. SA, H-JK, and CZ designed, synthesized, and analyzed the compounds; KS, H-JK, CS, and GJ defined screening strategy and selected compound libraries; CS, CB, MG, and BB performed compound screening. KB purified and tested the infectivity of AAV particles; FC performed molecular modeling analyses and phosphatidylserine exposure experiments; CS, ML, and H-GK designed the experiments and prepared the manuscript; ML, CS, JN-G, and H-GK revised and all authors approved the final manuscript.

### Conflict of interest statement

CB, MG, GJ, BB, SA, CZ, and CS were employed by JADO during the study. This company is no longer active, and the authors have no current affiliation to it. The remaining authors declare that the research was conducted in the absence of any commercial or financial relationships that could be construed as a potential conflict of interest.

## Footnotes

^1^Calculated via chemicalize.com.
