# Supplementary material for: Lipidomimetic Compounds Act as HIV-1 Entry Inhibitors by Altering Viral Membrane Structure
Source: Front Immunol. 2018 Sep 4;9:1983. doi: 10.3389/fimmu.2018.01983 (PMC6131562; doi:10.3389/fimmu.2018.01983)
Supplement: Supplementary file 1 [file Data_Sheet_1.PDF]

Supplementary Material

Lipidomimetic Compounds Act as HIV-1 Entry Inhibitors by Altering Viral Membrane Structure

Jon Ander Nieto, Bärbel Glass, Carmen Bunn, Matthias Giese, Gary Jennings, Beate Brankatschk, Sameer Agarwal, Kathleen Börner, F.-Xabier Contreras, Hans-Joachim Knölker, Claudia Zankl, Kai Simons, Cornelia Schroeder, Maier Lorizate* & Hans-Georg Kräusslich*

*Correspondence: Dr. Maier Lorizate, maier.lorizate@ehu.eus and Prof. Hans-Georg Kräusslich, hans-georg.kraeusslich@med.uni-heidelberg.de

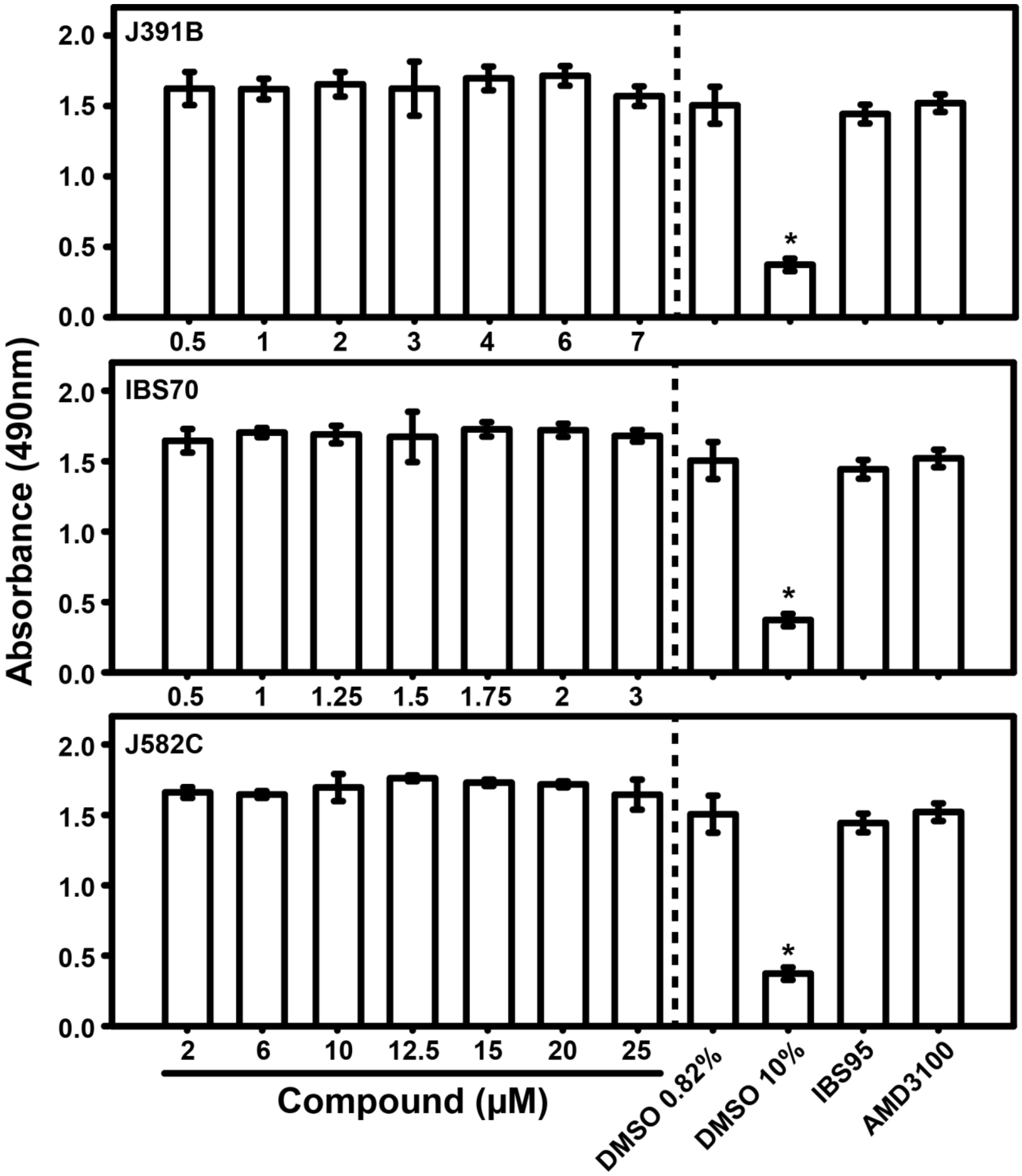

Figure S1. MTT test for cytotoxicity of lipidomimetics at different concentrations. TZM-bl cells were incubated for 2 h in the presence of the compounds. Concentration ranges of J391B, IBS70 and J582C are depicted in each panel. DMSO (0.82 %), IBS95 (7 µM) and AMD3100 (0.5 µM) served as controls and treatment with 10 % DMSO served as toxicity control. Data represent the mean ± SD of four replicates. * represents a significant ($p < 0.01$) decrease when compared to the DMSO control.

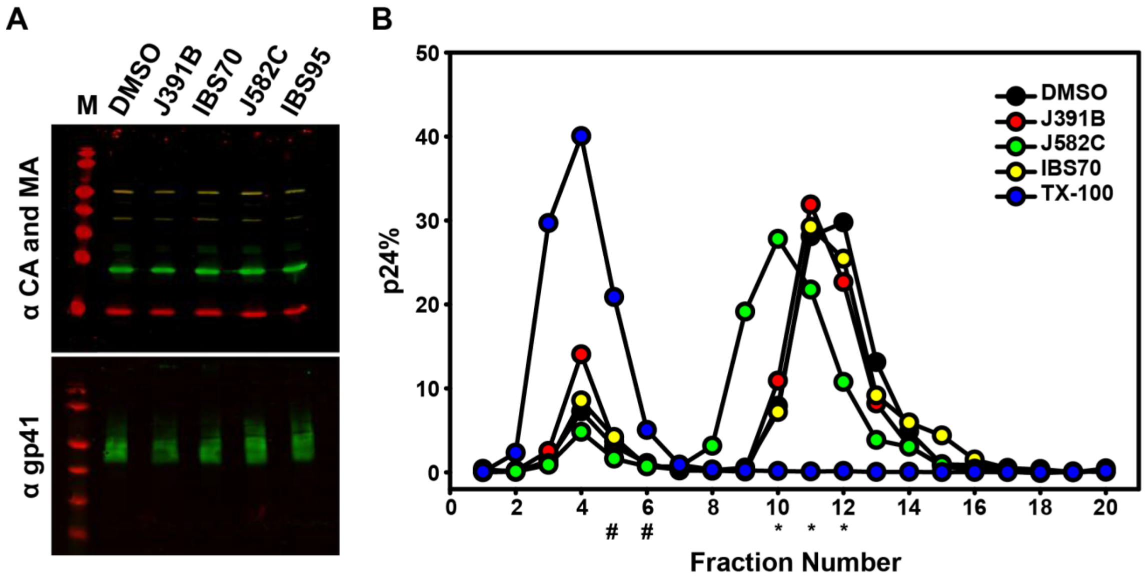

Figure S2. Influence of lipidomimetics on HIV-1 particle stability and density.

(A) Virus stability. Purified HIV-1 (3 µg of CA) was treated with 6 µM J391B, 2 µM IBS70, 20 µM J582C, 7 µM IBS95 or DMSO (0.35%) for 30 min at 37ºC. Subsequently, particles were recovered by ultracentrifugation and analyzed by Western blot using antisera against CA (green) and MA (red) and against the HIV-1 trans-membrane glycoprotein gp41.

(B) Virus buoyant density. HIV-1 (1 µg of CA) was treated with 6 µM J391B, 2 µM IBS70, 20 µM J582C, 0.5% TX-100 or DMSO (0.35%) as in panel A and subsequently subjected to equilibrium density gradient centrifugation. Gradient fractions were collected from the top and virus amounts were quantified by p24 ELISA.

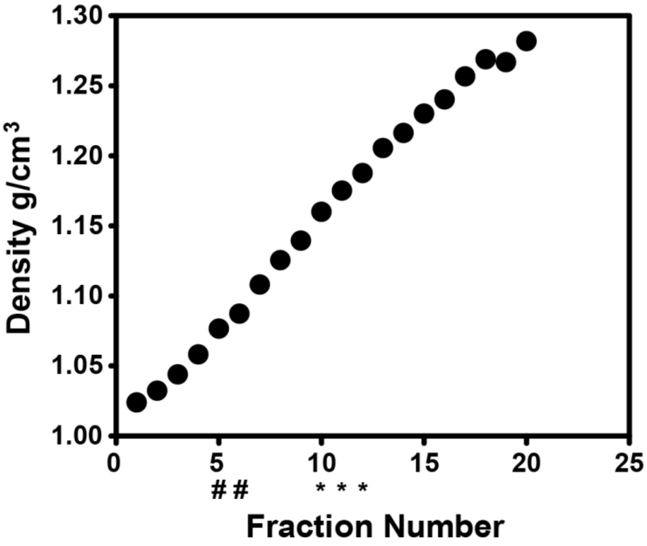

Figure S3. Density gradient fraction-density measured by refractometer.

Sucrose gradients were run and refractive index of the fractions was measured by refractometer. Fraction density was calculated. Asterisks * represent the location of virus and # the location of soluble capsid in the gradient.

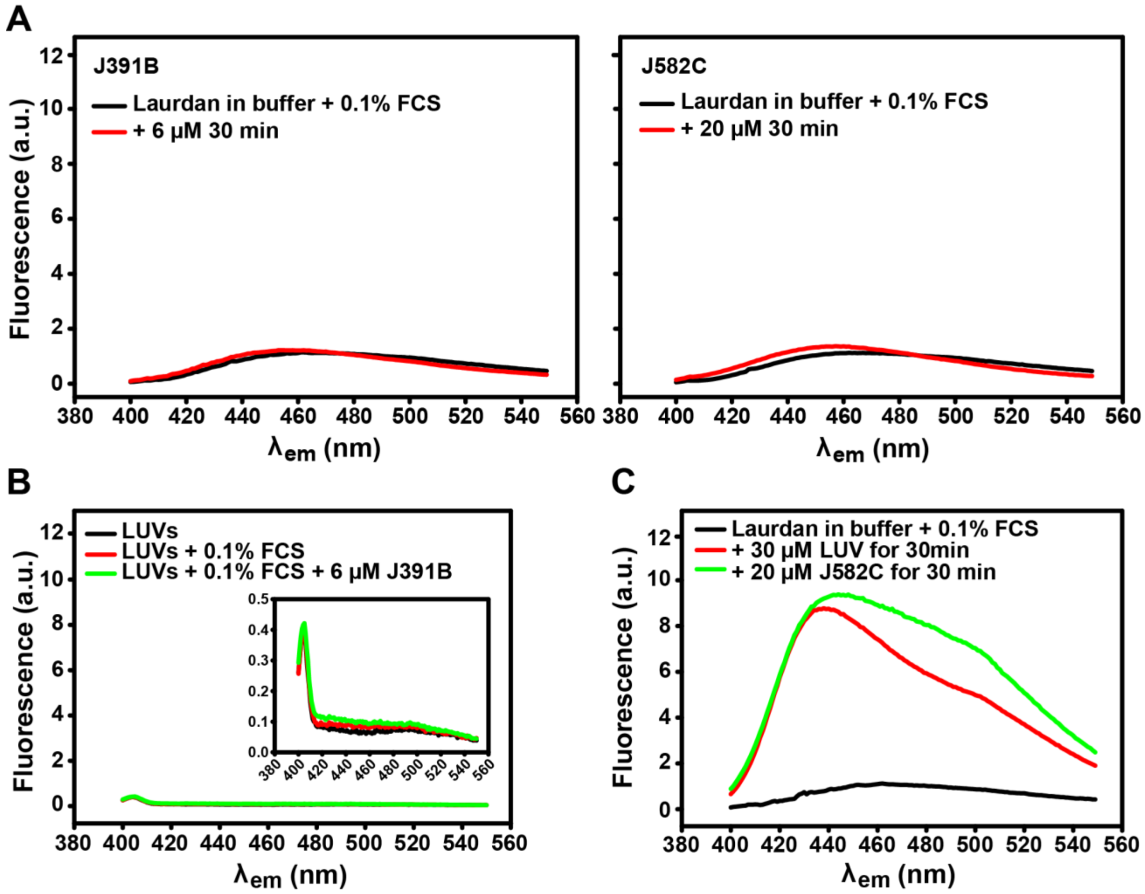

Figure S4. Control experiments.

(A) J391B (left) and J582C (right) do not influence base-line laurdan fluorescence in buffer.

(B) LUVs in the absence of laurdan, with or without J391B produce no fluorescence signal.

(C) Laurdan in buffer and in the presence of J582C has no fluorescence signal, but once liposomes are added laurdan fluorescence appears, as well as the fluorescence change caused by the compound.

Table 1: Overview of plasmids and virus strains used in this study

Constructs and viruses		Reference
pCHIV	HIV-1 proviral plasmid generating non-infectious HIV-like particles	Lampe et al. 2007
pNL4-3	HIV-1 proviral plasmid generating infectious particles	Adachi et al. 1986
pMM310	Plasmid encoding the Vpr-BlaM fusion protein	Münk et al. 2002
HIV-1_{NL4-3}	Infectious HIV-1 strain, CXCR4-tropic	Adachi et al. 1986
HIV-1 Vpr-BlaM	Infectious NL4-3 strain carrying BlaM within virus particles	Cavrois et al. 2002
MLV-Env	Friend ecotropic Murine Leukemia Virus glycoprotein	Sherer et al. 2003
VSV-G	G glycoprotein of Vesicular Stomatitis Virus	Emi et al. 1991
AAV2	Adeno-associated Virus	Grimm 2002
